# Bioaugmentation in anaerobic digesters: a systematic review

**DOI:** 10.1186/s13068-026-02746-6

**Published:** 2026-02-03

**Authors:** Mozhdeh Alipoursarbani, Jeroen Tideman, Mitzy López, Christian Abendroth

**Affiliations:** 1 Chair of Circular Economy, Brandenburg University of Technology Cottbus-Senftenberg, Cottbus, Germany; 2 Bioclear Earth B.V., Groningen, the Netherlands; 3 Universidad Nacional Autónoma de México, Mexico, Mexico

**Keywords:** Bioaugmentation, Anaerobic digestion, Biogas plants, Microorganisms, Microbiomes, Defined cultures

## Abstract

Bioaugmentation, the intentional introduction of specific microorganisms into anaerobic digestion (AD) systems, has shown promise in enhancing methane production and in mitigating stressful conditions, particularly in systems operating below optimal performance. This review presents a systematic literature review (SLR) of research on bioaugmentation in AD. This review identified and analysed studies meeting predefined eligibility criteria through a structured methodology involving research protocol, search, appraisal, synthesis, analysis, and reporting. A notable innovation of this review is its comprehensive critical comparison of different controls used in bioaugmentation studies, which has been inadequately addressed in previous literature. To facilitate the functional understanding, strains for bioaugmentation were grouped into the four phases of anaerobic digestion (hydrolysis, acidogenesis, acetogenesis and methanogenesis). A highly diverse set of microbes has been described for bioaugmentation, especially from the families *Clostridiaceae*, *Pseudomonadaceae* and *Syntrophomonadaceae*. Most works are related to hydrolysis. The few works that address acidogenesis are mostly related to dark fermentation. Several studies used methanogenic archaea as well as syntrophic acetate oxidising bacteria, despite the difficulties in culturing them. On the other hand, studies applying strains for acetogenesis were largely underrepresented. Especially works on syntrophic propionate and butyrate oxidation (SPO and SBO) were missing.

## Introduction

Fossil fuels continue to play a central role in supplying energy for both industrial and domestic purposes. However, this fuel presents two significant issues: its finite nature leading to eventual depletion and its environmental impact through greenhouse gas emissions, contributing to global warming. Thus, the development of renewable and environmentally friendly alternative energies is crucial to address these limitations.

In recent years, there has been an increasing trend towards the utilisation of anaerobic digestion (AD) for the production of biogas and renewable energy. AD offers a sustainable and environmentally friendly solution for the treatment of organic waste and the generation of valuable resources [1]. The utilisation of AD to convert organic waste into biogas is a highly effective approach to waste management [2]. AD is widely acknowledged as a technology for extracting energy (CH_4_) from organic waste through the action of various microbial communities within anaerobic conditions [3]. This process encompasses several stages including hydrolysis, acidogenesis, acetogenesis, and methanogenesis, with each phase being facilitated by specific microbial consortia [4]. The efficiency of AD depends on the metabolic activities and interactions of the microorganisms [5].

The alteration in microbial balance within bioreactors, often caused by the inhibition of certain groups of microorganism or the proliferation of others, is predominantly triggered by various inhibitory factors. These factors encompass elevated levels of inorganic toxicants like ammonium, phosphate, sulphate, and metal ions. Additionally, fluctuations in parameters such as temperature, pH, organic loading rate (OLR), and the resistance of feedstock to biodegradation contribute to decreased efficiency in AD. Among the proposed mitigation strategies are feedstock pretreatment (including ensilage) or dilution, implementation of multi-phase bioreactors, and precise control of temperature and pH, among others. While these strategies show promise, they may also extend the duration and consequently increase the cost of the AD process [6].

Given that the factors mentioned above induce shifts in microbial community dynamics, bioaugmentation emerges as a potential alternative strategy to address these limitations. In regard to anaerobic digestion, bioaugmentation involves the introduction of specific stress-resistant or efficient microorganisms into the underlying microbial community with the aim of bolstering its capacity to produce biomethane. This approach has demonstrated success in aerobic biodegradation scenarios, particularly in soil and wastewater, targeting contaminants typically resistant to degradation [7–9]. However, despite the advancements in AD technology, there are still significant challenges to overcome, particularly in the area of biodegradation enhancement or bioaugmentation. Bioaugmentation involves the addition of specific microbial consortia or enzymes to improve the breakdown of complex organic substrates and enhance the biogas production process. The successful implementation of bioaugmentation techniques holds great potential for optimising biogas yields, improving process stability/robustness, and facilitating the digestion of challenging feedstocks [10]. This literature review aims to explore the current state of AD for biogas production, with a specific focus on the challenges associated with bioaugmentation.

## Material and methods

### Data collection

**Fig. 1:**
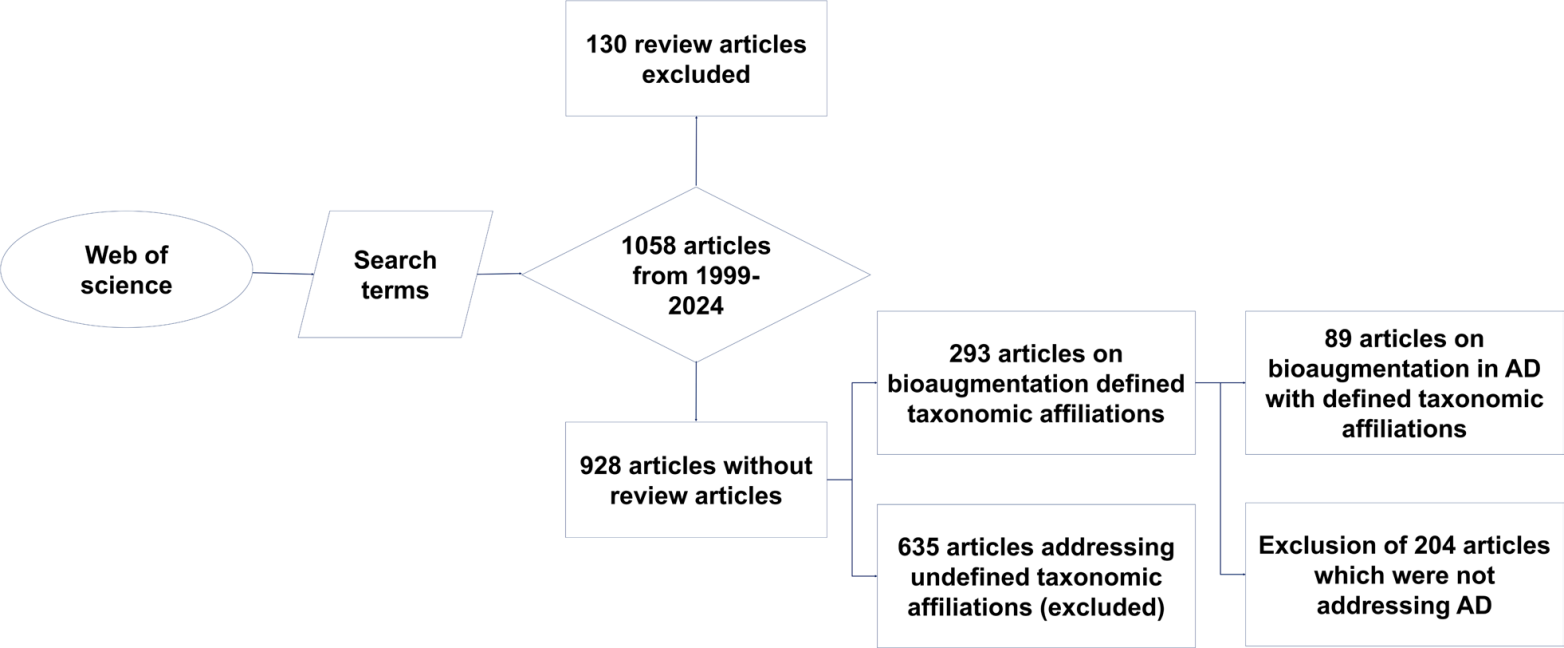
Flowchart depicting the methodology for conducting a systematic review using Web of Science

This review follows a systematic literature review (SLR) approach inspired by, Mengist et al. [11] aiming to ensure thoroughness, transparency, and reproducibility in identifying and analysing relevant literature. The systematic process utilised for conducting the search for relevant literature is illustrated in Fig. 1. A comprehensive examination of Clarivate's Web of Science (WoS) core collection was conducted to identify all publications related to anaerobic digestion, biogas production, and bioaugmentation. The search string "anaerobic OR biogas AND bio$augmentation" was applied across the database. The search was performed on March 31, 2024. To ensure reproducibility, the search was limited to peer-reviewed research articles published in English, excluding review articles, short communications, conference papers, book chapters, and other non-research documents. This yielded a total of 1058 documents. The parsing and analysis of the WoS corpus were carried out using the bibliometrix package in R as shown in Fig. 3. Subsequently, 130 review articles were excluded from the analysis, resulting in 928 articles for further examination. Among these articles, 293 specifically focused on bioaugmentation and included defined taxonomic affiliations. Within this subset of bioaugmentation papers, 89 articles specifically addressed bioaugmentation in the context of anaerobic digestion, with taxonomic affiliations also being defined. In total, 635 articles were excluded due to the lack of taxonomic affiliations, with a subset of these focusing on anaerobic digestion.

## Results and discussion

### Exclusion criteria

**Fig. 2:**
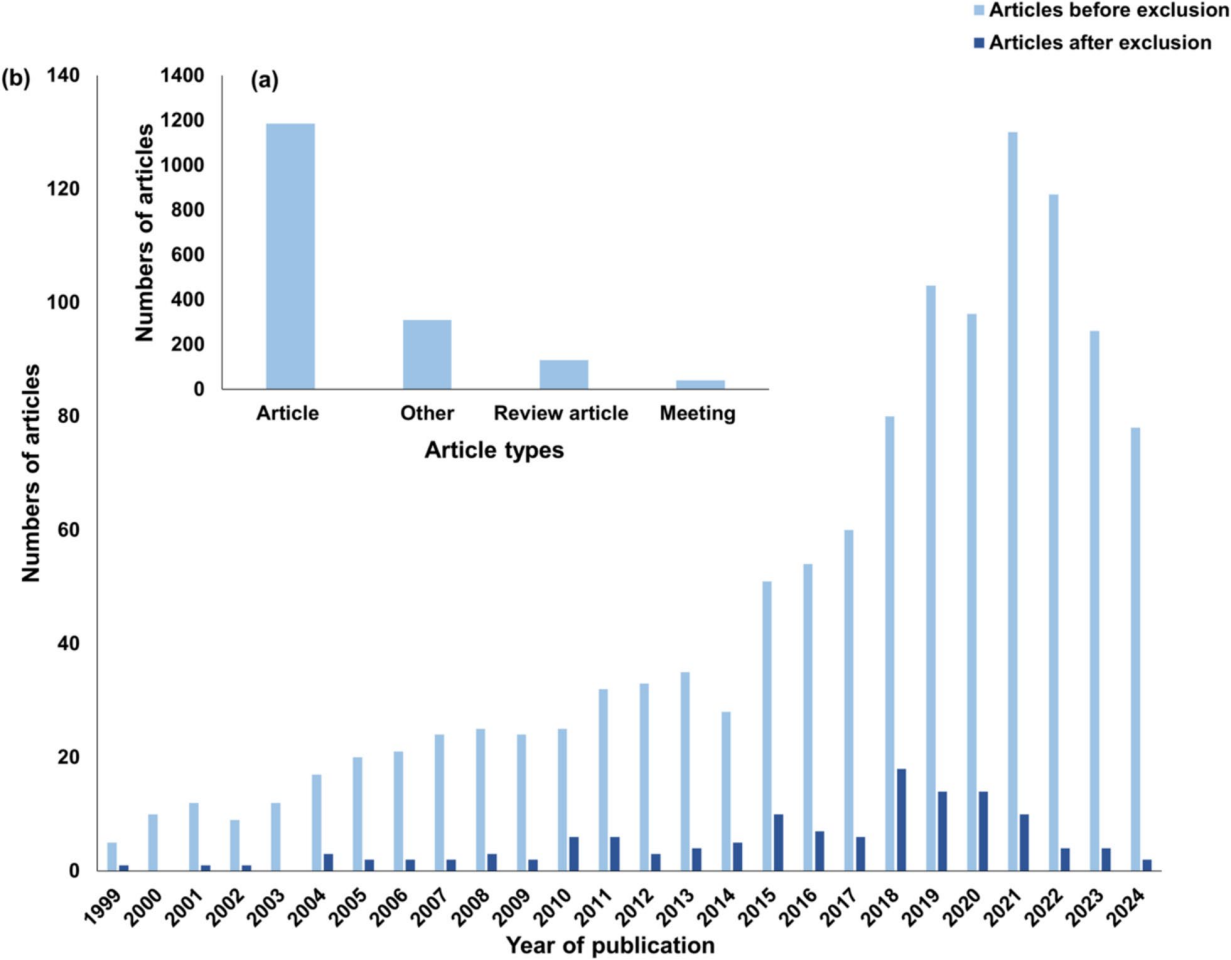
Article types and number of publications: article types of raw data (**a**); number of publications per year before and after the application of exclusion criteria (**b**)

The distribution of various types of articles is displayed in Fig. 2 (a). The predominant type of article identified was research papers. This finding suggests that researchers have dedicated significant efforts to investigating and contributing new knowledge in the subject area. Although bioaugmentation in the context of anaerobic digestion is a young field of research (beginning in 1999), there is already a considerable amount of review articles (130). Web of Science groups several articles into “Other” and “Meetings”. Considering that after applying the exclusion criteria, relatively few articles remained, it was decided to also take into account “Other” articles and as well articles from “Meetings”. Recently, it has been highlighted as a problem that many review articles are currently reviewing other review articles [12]. In agreement with this finding, all review articles were excluded from the reviewed set of articles. Nevertheless, the article should be differentiated from previous review articles in order to better emphasise the importance of the article. This has been detailed in Sect. “Differentiation from existing review articles”.

Beginning with a few items in 1999 and the early 2000 s, the number of items increased and now indicates exponential growth (Fig. 2b). This trend indicates a significant increase in the attention and interest of the scientific community towards this topic.

**Fig. 3:**
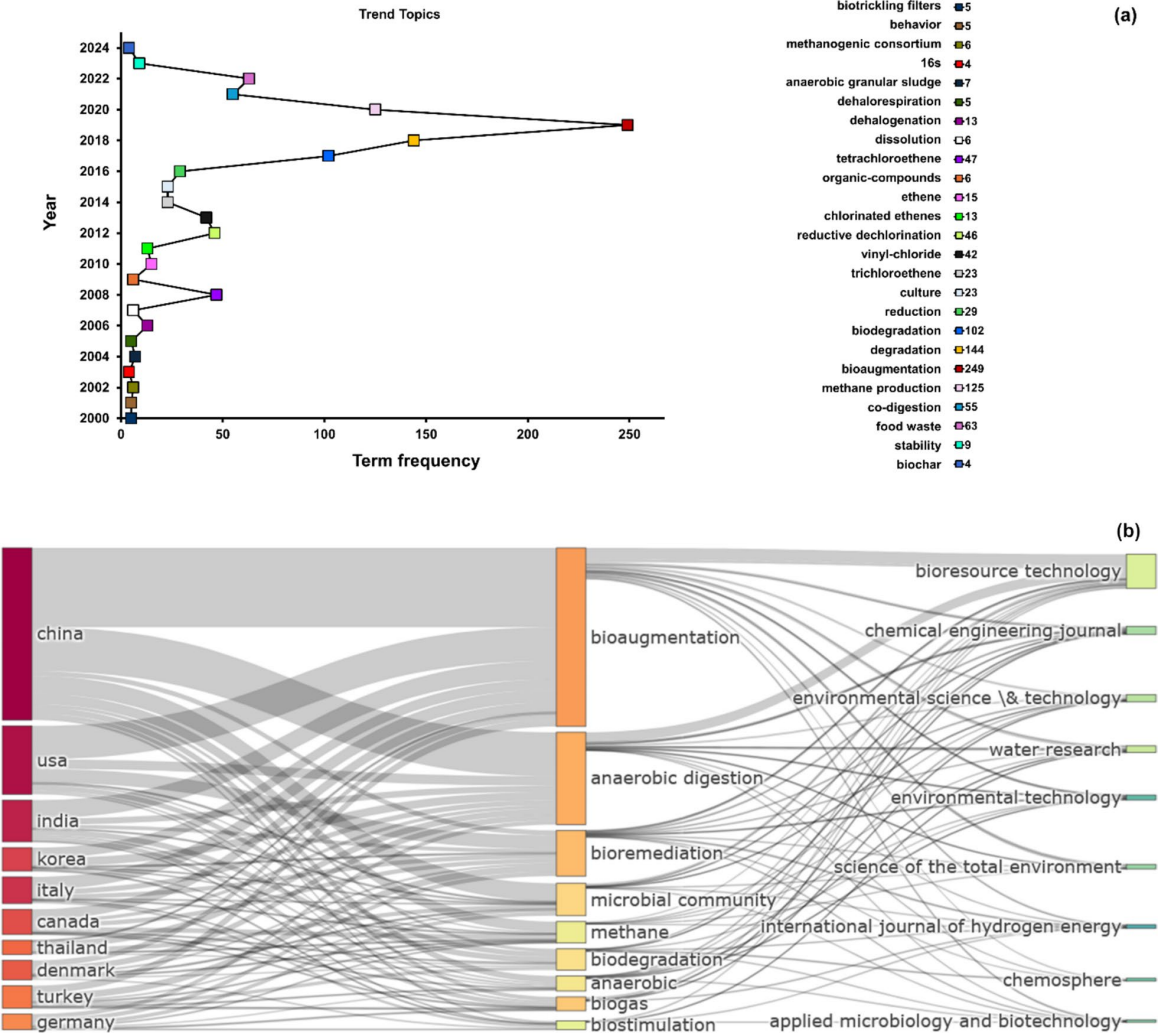
Key word analysis, journals and geographic distribution: **a** frequency of keywords from the years 2000 to 2024. The right side displays the ranking of each term by frequency, reflecting their prominence in the literature; **b** Sankey diagram illustrating the prominent countries, popular keywords, and frequently cited journals in the field. Both visualisations were prepared using the R package *bibliometrix*

Important keywords, their frequency, important journals as well as geographical distribution are shown in Fig. 3. Analysing the occurrence of important terms, it appears that the focus of interest was shifting over the years (Fig. 3a). The terms are ranked by frequency on the right side, reflecting their prominence in the literature. The connections between terms show the evolving research focus over time, revealing how topics have shifted as the field has developed. The early phase of research, represented by the leftmost terms, shows a strong focus on chemical processes involving chlorinated compounds. Terms such as "*dehalogenation*", "*tetrachloroethene*", "*chlorinated ethenes*", "*reductive dechlorination*", and "*vinyl chloride*" appear frequently and are closely connected. This suggests that early studies were primarily concerned with understanding and mitigating the environmental and health impacts of specific chlorinated organic pollutants. As research progressed, there was a noticeable shift towards microbial processes and biodegradation mechanisms. Terms such as "*culture*", "*biodegradation*", and "*reduction*" begin to appear, indicating that researchers started focusing on biological approaches for breaking down these pollutants. This shift reflects an increased interest in utilising natural microbial communities and bioaugmentation to enhance pollutant degradation, which is evident from the term *bioaugmentation*, one of the most frequently occurring topics in the graph. In more recent years, the focus has expanded to encompass broader environmental applications and sustainable waste management practices. Terms like "*methane production*", "*co-digestion*", "*food waste*", and "*stability*" suggest a growing emphasis on integrating waste management practices with environmental sustainability goals. This trend indicates a movement towards addressing not just pollutant removal, but also harnessing by-products (such as methane) in the process, thereby contributing to a circular economy approach.

The frequency data highlight some core topics that have sustained attention over time. "*Bioaugmentation*" (249 occurrences) and "*degradation*" (144 occurrences) are particularly prominent, emphasising the consistent research interest in enhancing microbial communities to break down pollutants effectively. The significant frequency of "*biodegradation*" (102 occurrences) and "*methane production*" (125 occurrences) further underscores a dual focus on both pollutant degradation and energy/resource recovery.

**Fig. 4:**
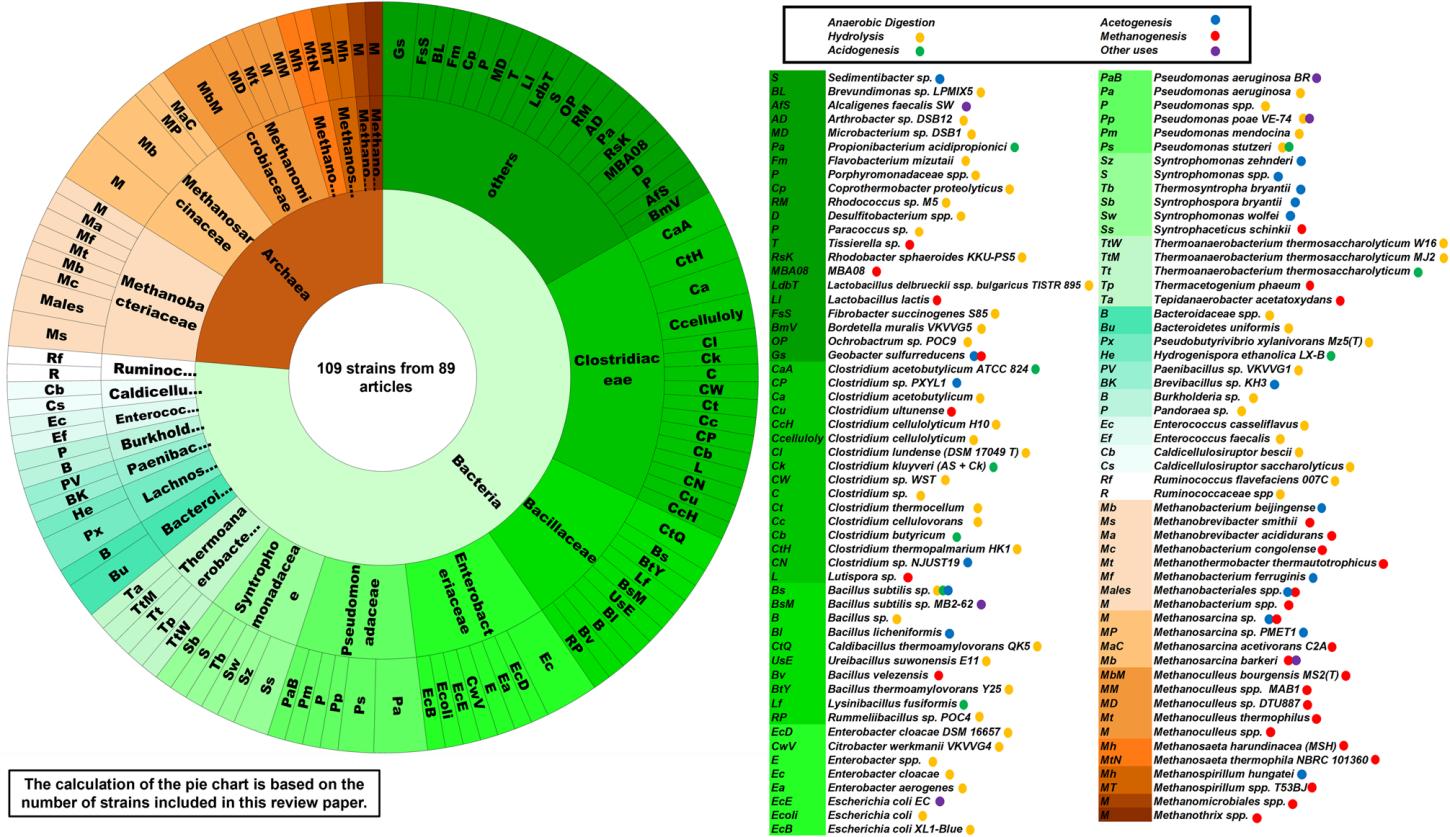
Pie chart indicating the diversity and frequency of microbial strains used for bioaugmentation in anaerobic digestion systems. In the figure and throughout the study, the affiliations *Methanothrix* and *Methanosaeta* have both been used. It must be noted that more recent works use *Methanothrix* instead of *Methanosaeta*, as *Methanosaetaceae* were renamed to *Methanotrichaceae*

Figure 3b provides a comprehensive overview of research trends in environmental biotechnology over nearly two decades. It highlights the diverse journals through which knowledge has been disseminated and illustrates the geographical distribution of research efforts. The figure effectively portrays the global landscape of contributions, emphasising the interconnectedness of leading countries, key research topics, and significant scientific publications in the field. Using a Sankey diagram, it highlights how major research areas such as "bioaugmentation", "anaerobic digestion", "bioremediation", and "microbial community" studies are distributed across various countries and published in specific journals. China and the USA are identified as the most significant contributors, with substantial research outputs across multiple topics, followed by other active countries like India, Korea, and Italy. The diagram reveals that *Bioresource Technology*, *Environmental Science & Technology*, and *Water Research* are among the top journals publishing these studies, with *Bioresource Technology* standing out as a primary publication venue across diverse topics. The interconnected flows in the diagram emphasise how different countries focus on similar research areas and target common journals, creating a cohesive international research network. This visualisation effectively captures the interdisciplinary and collaborative nature of environmental biotechnology research, underscoring the global commitment to advancing sustainable biotechnological solutions (Fig 4).

### Differentiation from existing review articles

While numerous review articles have been published on the topic of anaerobic digestion and bioaugmentation, this systematic review distinguishes itself by addressing critical gaps in the current literature. Many existing reviews provide comprehensive overviews of anaerobic digestion processes and the general application of bioaugmentation. However, they often lack a detailed analysis of the different control conditions under which bioaugmentation is implemented, and they do not adequately differentiate the specific roles of various microorganisms at each step of the anaerobic digestion process.

In anaerobic digestion, the microbial consortia involved are diverse and perform distinct functions at various stages, such as hydrolysis, acidogenesis, acetogenesis, and methanogenesis. While some reviews touch on the role of microorganisms in enhancing specific steps of bioprocesses, there remains a lack of comprehensive discussion on their effectiveness across various operational conditions. This review fills this gap by systematically identifying key microorganisms involved in different bioprocess steps and providing insights into how their effectiveness can vary under different substrates, environmental settings, and reactor configurations. By offering a more nuanced understanding of these factors, this work contributes to refining bioaugmentation strategies for diverse operational contexts. This review aims to build upon previous works by systematically analysing the impact of various control conditions, such as pH, temperature, and inoculum sources, on the effectiveness of bioaugmentation. While there have been studies addressing aspects of bioaugmentation, our approach differentiates the microbial roles and optimises conditions for each stage of anaerobic digestion in a comprehensive manner. By focussing on taxonomy differentiating the microbial roles and optimising conditions for each stage of anaerobic digestion, this review provides a more targeted approach to improving the efficiency and effectiveness of bioaugmentation in anaerobic digester plants.

### Controls used in bioaugmentation experiments

In this section, the focus lies on the various measures implemented to control the growth and activity of added microbes in the context of bioaugmentation experiments. These controls are crucial for ensuring reliable and accurate results while studying the potential of bioaugmentation for enhancing biological processes. The initial aim of this section was to highlight, which controls were used to demonstrate that bioaugmentation resulted in increased biogas levels. However, it turned out that the diverse setups of different experiments make the categorisation difficult, especially since not all experiments aimed to produce methane. Originally, it was planned to divide all experiments into cases with (1) no control; (2) control without adding additional bioaugmentation strains; (3) controls, where autoclaved strains were added; (4) positive controls with strains, which are known to give positive results. However, it turned out that the applied search sometimes resulted also in some extraordinary approaches, which were dealing with rather unusual scenarios (e. g. the screening for new strains for bioaugmentation or MFCs. For these (5) extraordinary approaches a fifth type of control has been defined, which is further referred to as “other”. All found articles have been assigned to these five groups of controls (Tables 1, 2, 3 and 4). In several articles, insufficient control experiments were observed. In several articles, no control was used. Usually, there is only one type of control applied (control group 2, 3 or 4). On few occasions, two types were applied. In none of the studies, all three types of controls were applied.

**Table 1:** Summary of bioaugmentation studies targeting the hydrolysis phase

Authors	Microbial strain(s) used	Reactor type and scale	Type of control	Feedstock	Type of endpoint	ISR
Zhang et al. [20]	*Thermoanaerobacterium thermosaccharolyticum W16*	100 mL serum (batch)	2	Corn stover	AD-CH_4_	0.04*
Nielsen et al. [22]	*Not clear*	Not clear	Not clear	Not clear	AD-CH_4_	Not clear
Barua et al. [23]	*Citrobacter werkmanii VKVVG4; Bordetella muralis VKVVG5; Paenibacillus sp. VKVVG1*	1 L reactor bottles (batch)	4, 6	Water hyacinth plant and cow dung	AD-CH_4_	0.01*
Peng et al. [24]	*Clostridium cellulolyticum*	Not clear	3, 4	Cellobiose and wheat straw	AD-CH_4_	2
Ecem Öner et al. [25]	*Clostridium thermocellum*	100 mL serum bottles (batch)	4	Wheat straw	AD-CH_4_	1.00*
Ozbayram et al. [26]	*Bacteroidaceae spp.; Porphyromonadaceae spp.; Ruminococcaceae spp.*	100 mL bottle (batch)	-	Wheat straw from cow and goat rumen fluid	Non-AD	N/A
Sinha et al. [27]	*Microbacterium sp. DSB1; Arthrobacter sp. DSB12*	2 L heavy-duty vacuum bottle (batch)	4	Lantana camara	AD-CH_4_	Not clear
Shanmugam et al. [28]	*Clostridium sp. WST*	100 mL serum bottle (batch)	2	Xylan	Non-AD	N/D
Kavitha et al. [29]	*Bacillus sp.*	250 mL conical flask (batch)	4	Microalga	AD-CH_4_	7.00*
Mulat et al. [30]	*Caldicellulosiruptor bescii*	120 mL bottles (batch)	4	Sewage sludge and food waste	AD-CH_4_	0.06*
Vidmar et al. [31]	*Pseudobutyrivibrio xylanivorans Mz5T*	CSTRs with 500 ml volume	3, 4	Microalgae	AD-CH_4_	1.63*
Costa et al. [32]	*Clostridium cellulolyticum; Caldicellulosiruptor saccharolyticus; Clostridium thermocellum*	1 L bottle (batch)	4, 6	Raw poultry litter waste	AD-CH_4_	0.37*
Čater et al. [21]	*Ruminococcus flavefaciens 007 C; Pseudobutyrivibrio xylanivorans Mz5T; Fibrobacter succinogenes S85; Clostridium cellulovorans*	1 L reactors (batch)	2, 3	Brewery	AD-CH_4_	0.24*
Senko et al. [33]	*Clostridium acetobutylicum; Pseudomonas sp.; Enterococcus faecalis*	120 mL reactor (batch)	4	Aspen sawdust, pine sawdust, Jerusalem artichoke stems, chicory stems, beet pulp, bagasse (sugarcane residue)	AD-CH_4_	1.00*
Sarkar et al. [34]	*Enterobacter sp.; Pandoraea sp.; Burkholderia sp.*	100 mL serum vials (batch)	4	Crude oil	Non-AD	N/A
Loureiro et al. [35]	*Pseudomonas aeruginosa; Pseudomonas stutzeri; Pseudomonas mendocina*	125 mL glass bottles (batch)	2	B10 diesel oil	Non-AD	N/A
Guo et al. [36]	*Paracoccus sp.*	500 mL glass bottles (batch)	4	Phytoremediation residues	AD-CH_4_	0.003*
Puyol et al. [37]	*Desulfitobacterium spp.*	2.5 L batch	5	Granular sludges	Non-AD	N/A
Tartakovsky et al. [38]	*Rhodococcus sp. M5*	UASB reactor	4	Granular biofilm sludge	Non-AD	N/A
Cayetano et al. [41]	*Bacteroidetes uniformis; Clostridium sp.*	150 mL serum bottles (batch)	4	Waste-activated sludge	AD-CH_4_	8.07*
Poszytek et al. [39]	*Rummeliibacillus sp. POC4*	1 L glass bottles	4	Sewage sludge	AD-CH_4_	1.00*
Poszytek et al. [40]	*Ochrobactrum sp. POC9; Brevundimonas sp. LPMIX5*	1 L bottles (batch)	4	Sewage sludge	AD-CH_4_	1.00*
Li et al. [42]	*Flavobacterium mizutaii; Pseudomonas sp.*	100 ml flasks	4	Swine wastewater	AD-CH_4_	-
Lin et al. [43]	*Pseudomonas aeruginosa*	1.5 L MBBRs reactor	2, 6	Synthetic wastewater	Non-AD	N/A
Lü et al. [44]	*Coprothermobacter proteolyticus*	Sequenced batch reactor	4	Granular sludge	AD-CH_4_	0.01*
Cirne et al. [45]	*Clostridium lundense DSM 17049 T*	100 cm^3^ serum bottles (batch)	3, 4	Restaurant lipid-rich waste	AD-CH_4_	1.35*
Xiao et al. [46]	*Clostridium thermopalmarium HK1; Caldibacillus thermoamylovorans QK5*	CSTRs with 5 L volume	4, 6	Food waste	AD-CH_4_	2.00
Mazzurco Miritana et al. [47]	*Orpinomyces sp.; Neocallimastix sp.*	120 mL serum bottles (batch)	4	Shrimp processing waste	AD-CH_4_	0.19*
Huang et al. [48]	*Thermoanaerobacterium thermosaccharolyticum MJ2*	55 mL serum bottles (batch)	4	Sugarcane bagasse	Non-AD	N/A
Camargo et al. [49]	*Enterococcus casseliflavus*	500 mL flasks (batch)	4	Citrus peel waste	AD-H_2_	0.50*
Kumar et al. [50]	*Escherichia coli XL1-Blue; Enterobacter cloacae DSM 16657*	225 mL bath fermentation (batch)	4	Beverage industrial wastewater	AD-H_2_	0.06*
Laocharoen et al. [51]	*Rhodobacter sphaeroides KKU-PS5; Lactobacillus delbrueckii ssp. bulgaricus TISTR 895*	Not clear	Not clear	Not clear	AD-H_2_	Not clear
Sharma & Melkania [52]	*Escherichia coli; Bacillus subtilis; Enterobacter aerogenes*	500 mL bottles (batch)	4	Organic fraction of municipal solid waste	AD-H_2_	1.04*
Jung [53]	*Clostridium cellulolyticum H10*	Not clear	Not clear	Not clear	AD-CH_4_	Not clear
Kovács et al. [54]	*Caldicellulosiruptor saccharolyticus; Enterobacter cloacae*	5 L CSTR	4	Anaerobic sludge	AD-CH_4_	0.26*
Ács et al. [55]	*Enterobacter cloacae*	5 L CSTR reactors (batch)	4	Anaerobic sludge	AD-CH_4_	0.03*
Morales-Martínez et al. [56]	*Clostridium acetobutylicum*	120 mL glass bottles (batch)	4	Agave biomass	AD-H_2_	0.004*
Wang et al. [57]	*Ureibacillus suwonensis E11; Clostridium thermopalmarium HK1; Bacillus thermoamylovorans Y25; Caldibacillus thermoamylovorans QK5*	400 mL bottles (batch)	4, 6	Food waste	AD-CH_4_	2.00

^*^ ISR values were calculated as the ratio of mass of inoculum and mass of substrate, as suggested by Rakić et al. [100]. Both, the mass of inoculum and the mass of substrate was normalised by converting into weight of volatile solids. Values marked as N/A correspond to studies where ISR was not applicable or could not be determined due to non-AD endpoints or unavailable data. Types of controls, which have been used in publications on bioaugmentation have been assigned as (1) no control, (2) control without adding additional bioaugmenting strains, (3) controls, where autoclaved strains were added, (4) positive controls with strains, which are known to give positive results, (5) negative controls with strains, which are known to give ineffective results other, (6) (approaches with rather unusual scenarios)

**Table 2:** Summary of bioaugmentation studies targeting the acidogenesis phase

Authors	Microbial strain(s) used	Reactor type and scale	Type of control	Feedstock	Type of endpoint	ISR
Yang et al. [58]	*L. hydrogenispora ethanolica* LX-B	120 mL bottles (batch & semi-cont.)	4	Fresh potatoes	AD-H_2_	Batch cultivation: 0.01* Repeated batch cultivation: 0.11*
Atasoy & Cetecioglu [59]	*Clostridium butyricum*	2000 mL ASBR	4	cheese production industry wastewater	AD-VFAs production	Granular seed sludge: 1.42* *Clostridium butyricum*: 0.10*
Dams et al. [60]	*Clostridium acetobutylicum* ATCC 824	250 mL serum bottles	4	Residual glycerol generated from soybean and cow fat	AD-H_2_	Goat rumen liquid: 0.05* Flocculent sludge: 0.03* Granular sludge: 0.06*
Goud et al. [61]	*Bacillus subtilis*; *Pseudomonas stutzeri*; *Lysinibacillus fusiformis*	1.2 L reactors	4	Composite wastewater	AD-H_2_ and VFAs production	5 g/L COD: 0.08* 10 g/L COD: 0.04* 20 g/L COD: 0.02* 30 g/L COD: 0.01*
Wang et al. [62]	*Clostridium acetobutylicum ATCC 824*	1.48 m^2^ batch reactor	2	molasses	AD-H_2_	0.20*
Zheng et al. [63]	*Propionibacterium acidipropionici*	500 mL bioreactor	4	kitchen waste	AD-propionic acid production	BD10: 0.08* BD30: 0.24* BD50: 0.40* BD70: 0.56*
Liu et al. [64]	*Thermoanaerobacterium thermosaccharolyticum*	200 mL bottles (1st stage of 2-stage AD)	4	Food waste	AD-H_2_	1.00
Zagrodnik et al. [65]	*Clostridium kluyveri*	120 mL serum bottles	4	Waste-activated sludge	Non-AD	N/A

^*^ ISR values were calculated as the ratio of mass of inoculum and mass of substrate, as suggested by Rakić et al. [100]. Both, the mass of inoculum and the mass of substrate was normalised by converting into weight of volatile solids. Values marked as N/A correspond to studies where ISR was not applicable or could not be determined due to non-AD endpoints or unavailable data. Types of controls, which have been used in publications on bioaugmentation have been assigned as (1) no control, (2) control without adding additional bioaugmenting strains, (3) controls, where autoclaved strains were added, (4) positive controls with strains, which are known to give positive results, (5) negative controls with strains, which are known to give ineffective results other, (6) (approaches with rather unusual scenarios)

**Table 3:** Summary of bioaugmentation studies targeting the acetogenesis phase

Authors	Microbial strain(s) used	Reactor type and scale	Type of control	Feedstock	Type of endpoint	ISR
Wang et al. [70]	*Syntrophospora bryantii; Sedimentibacter sp.; Thermosyntropha bryantii; Methanosarcina sp.; Methanobacterium ferruginis*	Not specified	4	Glucose and molasses wastewater	Non-AD	0.10*
Huang et al. [71]	*Clostridium sp. NJUST19*	250 mL serum bottles (batch)	4	Waste-activated sludge	Non-AD	Thermophilic: 1.00*, mesophilic: 0.25*
Shao et al. [72]	*Methanobacteriales; Syntrophomonas*	Anaerobic sequencing batch reactor	4	Seed sludge	AD	-
Tale et al. [73]	*Methanospirillum hungatei; Methanobacterium beijingense*	UASB reactor	2	Propionate	AD-CH_4_	0.40*
Akila & Chandra [74]	*Clostridium sp. PXYL1; Methanosarcina sp. PMET1*	Small batch bottles [50–100 ml]	4,6	Cattle manure	AD-CH_4_	-
Cavaleiro et al. [75]	*Syntrophomonas zehnderi*	100 ml Anaerobic bottles	4	Oleate	AD	-
Wang et al. [76]	*Syntrophomonas wolfei; Geobacter sulfurreducens*	MFC	4,6	Sodium butyrate	MFC	-
Tian et al. (101)	*Methanoculleus sp.*	2.3 L CSTR (batch)	2	Animal manure and food industrial organic waste	Non-AD (ammonia toxicity)	0.18*
Larsen et al. [102]	Proteiniphilum acetatigenes	300 ml glass bottles	4	Sewage sludge	Non-AD	1.36*

^*^ ISR values were calculated as the ratio of mass of inoculum and mass of substrate, as suggested by Rakić et al. [100]. Both, the mass of inoculum and the mass of substrate was normalised by converting into weight of volatile solids. Values marked as N/A correspond to studies where ISR was not applicable or could not be determined due to non-AD endpoints or unavailable data. Types of controls, which have been used in publications on bioaugmentation have been assigned as (1) no control, (2) control without adding additional bioaugmenting strains, (3) controls, where autoclaved strains were added, (4) positive controls with strains, which are known to give positive results, (5) negative controls with strains, which are known to give ineffective results other, (6) (approaches with rather unusual scenarios)

**Table 4:** Summary of bioaugmentation studies targeting the methanogenesis phase

Authors	Microbial strain(s) used	Reactor type and scale	Type of control	Feedstock	Type of endpoint	ISR
Zhang et al. [10]	*Geobacter sulfurreducens*	100 mL serum bottles (batch)	4	Sodium acetate	AD-CH_4_	9.40*
Yang et al. [81]	*Methanosarcina barkeri; Methanobrevibacter smithii; Syntrophaceticus schinkii*	500 mL reactor (batch)	4, 5	Basal anaerobic (BA) medium	AD-CH_4_	2.00
Xiao et al. [80]	*Clostridium pasteurianum*	2 L bottle (batch)	4, 6	Granular sludge	AD-CH_4_	4.00*
Fotidis et al. [84]	*Clostridium ultunense spp.; Methanoculleus spp.; Methanoculleus bourgensis MS2*^*T*^	UASB reactors (batch)	5	Basal anaerobic (BAN) medium	Non-AD (ammonia toxicity)	N/A
Fotidis et al. 77)	*Methanomicrobiales spp.; Methanobacteriales spp.*	118 mL batch reactor	2, 6	Animal manure and organic waste	AD-CH_4_	2.00
Yan et al. [87]	*Methanoculleus sp. DTU887*	4.5 L CSTR reactors (batch)	4	Organic fraction municipal solid waste	AD-CH_4_	0.60
Yan et al. [88]	*Methanoculleus thermophilus sp.*	2.3 L CSTR reactors (batch)	4	Food waste	AD-CH_4_	0.10
Hua et al. [93]	*Methanosarcina acetivorans C2A; Methanosaeta thermophila NBRC101360*	140 mL batch	4	Cow manure	AD-CH_4_	0.40*
Savant et al. [94]	*Methanobrevibacter acididurans*	125 ml serum bottles	2	Distillery wastewater	AD-CH_4_	-
Li et al. [4]	*Methanosaeta*	anaerobic baffled reactor	2	Sucrose and Vanderbilt mineral medium	AD-CH_4_	1.7*
Town & Dumonceaux [95]	*Methanosarcina sp.*	100 mL syringe (batch)	2, 6	Thin stillage, cattle manure	AD-CH_4_	0.0008*

^*^ ISR values were calculated as the ratio of mass of inoculum and mass of substrate, as suggested by Rakić et al. [100]. Both, the mass of inoculum and the mass of substrate was normalised by converting into weight of volatile solids. Values marked as N/A correspond to studies where ISR was not applicable or could not be determined due to non-AD endpoints or unavailable data. Types of controls, which have been used in publications on bioaugmentation have been assigned as (1) no control, (2) control without adding additional bioaugmenting strains, (3) controls, where autoclaved strains were added, (4) positive controls with strains, which are known to give positive results, (5) negative controls with strains, which are known to give ineffective results other, (6) (approaches with rather unusual scenarios)

Comparing the “types of experiment” in Tables 1, 2, 3 and 4 it becomes clear that the Biochemical Methane Potential (BMP) assay is a common choice. This assay is a valuable method for determining the ultimate biodegradability and methane conversion yield of organic substrates. In this regard, the methane yield expresses the amount of methane per substrate. And the methane productivity relates to the amount of volume per time. [13] A critical parameter in the BMP assay is the inoculum-to-substrate ratio (ISR), which significantly influences the efficiency of anaerobic degradation, the relevance of the degradation test to full-scale digesters and the accuracy of the assay. Research has shown that a higher ISR can improve the ultimate practical methane yield, although the productivity might decrease. For example, a batch digestion test on microalgae found that an ISR of 2, compared to 1 and 0.33, resulted in the lowest methane productivity, ranging from 188 to 395 mL CH₄/g VS added across different microalga types [14]. The digestion of sunflower oil cake at an ISR of 3, compared to lower ratios, produced the highest methane yield [15]. However, at lower ISRs, while the maximum specific methane production rate was higher, the overall methane yield per weight of substrate was lower, as observed in BMP tests of maize at various ISRs [15]. This lower yield at low ISRs was linked to the accumulation of longer-chain acids within the system, which could inhibit methanogens, particularly due to the acetate produced during digestion at high substrate concentrations [16]. Increasing the ISR, which involves diluting the substrate, can help enhance practical methane yield. Most studies have focused on the impact of ISR on methane yield for single substrates, with limited documentation on its effects in co-digestion scenarios. Additionally, the source of inoculum is crucial, especially when dealing with complex substrate mixtures, due to the diverse microbial consortia involved. Calculating the ISR is important because it directly impacts the methane production efficiency and overall yield, which is illustrated in Tables 1, 2, 3 and 4. Different substrates produce varying methane outputs, which can be effectively assessed by considering the ISR in the BMP assay. It is hypothesised by the authors a low ISR, or usage of an inoculum source that is unsuitable for the substrate of choice can lead to false-positives on the effect of bioaugmentation.

The ISR values presented in Tables 1, 2, 3 and 4 show significant variability across different studies, reflecting diverse experimental setups and microbial additives. Analysing trends, ISR values can be grouped into three strata: low ISR (≤ 1), medium ISR (> 1 to ≤ 2), and high ISR (> 2). If no inhibitory concentration in VFA is reached, low ISRs results in higher methane productivity. However, incomplete conversion of VFA may result in low methane yield [17]. Studies with medium ISR ratios show improved and more consistent yields and the productivity is still considerably high. In contrast, studies with high ISR ratios present the highest methane yield, but low productivity. Therefore, the authors recommend an ISR, where both the methane yield and methane ratio are relatively high.

Holliger et al. [18] suggested an ISR between 2 and 4 for most applications, and ≥ 4 for easily degradable substrates to prevent VFA accumulation and inhibition. In this review only 34% of the studies meet the ISR threshold of > 1, and only 15% of the studies reached the recommended ISR of > 2. Among the entries, the highest ISR is reported by Zhang et al. [10], with a value of 9.4, achieved using a pure culture in 100 mL serum bottles aimed at biogas production. This indicates that the selected microbial strains and experimental conditions can greatly influence ISR outcomes. In contrast, studies such as Arkatkar et al. [19] report much lower ISR values, such as 0.01, due to the use of a pure culture in a microbial fuel cell (MFC) setup. The discrepancies in ISR values across authors can be attributed to differences in the type of cultures used (mixed vs. pure), the experimental design (batch vs. continuous systems), and the specific aims of the research, such as methane or hydrogen production. Therefore, while some ISRs suggest strong potential for biogas formation, others indicate challenges that may require further optimisation or different microbial approaches. Overall, identifying the most effective ISR for biogas production depends on the specific research context and microbial strains utilised.

### Manipulation of hydrolysis

The present work tries to distinguish applied microbes according to the different phases of anaerobic digestion (hydrolysis, acidogenesis, acetogenesis and methanogenesis). This separation is not always feasible, as there are overlaps. For example, some hydrolytic bacteria yield organic acids, which results in an overlap between hydrolysis and acidogenesis. This simultaneous involvement blurs the boundaries between the hydrolysis and acidogenesis phases. Similar challenges arise in other phases, such as acetogenesis, where certain microorganisms may contribute to both acidogenesis and acetate production, creating further complexities in differentiation. The difficulty of dividing found articles into the various phases of anaerobic digestion is also made clear in a work by Zhang et al. [20]. Zhang et al. [20] used the hydrolytic *Thermoanaerobacterium thermosaccharolyticum W16* mixed with undefined methanogenic granular sludge. It did not only improve hydrolysis, but also syntrophic relations, which are rather related to the later phases of anaerobic digestion. Nevertheless, the authors of the present study tried to distinguish the phases as clearly as possible, starting with hydrolysis. Hydrolysis contemplates the first stage of anaerobic digestion. During hydrolysis, complex organic compounds (e.g., carbohydrates, proteins, and lipids) will be transformed into simpler molecules, like sugars, long chains of fatty acids and amino acids due to the enzymatic attack made by different types of anaerobic microorganisms. During this stage, various obstacles may arise that limit the AD process, as well as the performance and adequate production of biogas.

The use of bioaugmentation within hydrolysis has been studied for various purposes with the overall goal of improving the efficiency and stability of the AD process, as shown in Table 1. The systematic literature search performed in the present work resulted in 33 articles, which were predominantly focused on the inoculation of hydrolytic microorganisms for bioaugmentation within the first stage of AD. Many of them are focussed on the improved degradation of fibre-rich material. For example, two of them highlighted the use of bioaugmentation to increase the methane yield from cattle manure and brewery spent grain. Hydrolytic organisms are promising here, as the mentioned substrates contain lignocellulosic biomass, which usually degrades very slowly and, therefore, the hydrolysis takes longer to complete (Nielsen et al., [21, 22]. In a similar approach, improved lignocellulose degradation has been shown with *Citrobacter* *werkmanii VKVVG4, Bordetella muralis VKVVG5* and *Paenibacillus sp. VKVVG1,* but for water hyacinth [23]. Another promising approach was presented by Peng et al. [24], who was able to enhance wheat straw hydrolysis and to improve the biochemical methane potential (BMP) from wheat straw due to the application of the cellulolytic anaerobic bacterium *Clostridium cellulolyticum*. The outcomes showed BMPs of 342.5 ml g^−1^ VS and 326.3 ml g^−1^ VS, representing a 13.0% and 7.6% increase, respectively, compared to the BMP without bioaugmentation, which was 303.3 ml g^−1^ VS. Similar to Peng et al., Ecem Öner et al. [25] worked on the degradation on wheat straw too. They used *Clostridium thermocellum* to enhance methane yield from lignocellulosic biomass by up to 39%. Ozbayram et al. [26] also worked with wheat straw as a substrate, enriching methanogenic communities from cow and goat rumen fluid and a biogas reactor. The dominant strains in the enriched cultures were *Bacteroidaceae spp*. (rumen) and *Porphyromonadaceae spp.* (reactor), with an increased abundance of *Ruminococcaceae spp.* (Firmicutes). Similarly, Sinha et al. [27] employed the cellulolytic strains *Microbacterium sp. DSB1* and *Arthrobacter sp. DSB12* for lignocellulose degradation of *Lantana camara*, achieving enhanced biogas production with methane yields of 57% and 60%, respectively. Based on the aforementioned articles it stands out that especially bacteria from the phylum Firmicutes are used abundantly to improve the degradation of lignocellulose. In this regard, another study by Shanmugam et al. [28] can be highlighted, which focused on the strain *Clostridium sp. WST.* After isolating it from mangrove sediments, this strain improved the degradation of lignocellulosic biomass degradation due to bioaugmentation in anaerobic digestion experiments.

Another article, where cellulolytic bacteria (*Bacillus sp.*) were applied too, has been presented by Kavitha et al. [29]. The work stands out as the experiment was not just focussed on improved hydrolysis from substrates within a given methanogenic digester. Instead, the substrate was pretreated before it was entered into the respective reactor. In this specific case, Kavitha et al. [29] investigated the impact of bacterial-based biological pretreatment on the liquefaction of *Chlorella vulgaris* microalgae before anaerobic biodegradation. The results show that pretreatment with cellulose-secreting bacteria increase the biomass stress index by 18% compared to the control group. The biomass stress index measures the physical and chemical stress experienced by the biomass during the pretreatment process, indicating the extent to which the biomass is broken down and made more amenable to subsequent processes. In this study, pretreatment significantly enhanced the biomass stress index, demonstrating its effectiveness in preparing Chlorella vulgaris for anaerobic biodegradation. Similar to Kavitha et al., Mulat et al. [30] and Vidmar et al. [31] tried to improve biomass pretreatment too. For this, Mulat et al. combined steam explosion (SE) and bioaugmentation, which significantly enhanced methane yield from birch. Compared to the untreated control, the yield increased up to 140%. Bioaugmentation with *Caldicellulosiruptor bescii* at lower dosages (2% and 5% of inoculum volume) showed the best methane improvement on day 50. Additionally, the microbial community analysis indicated an increase in abundance of key bacterial and archaeal communities, including the hydrolytic bacterium *Caldicoprobacter*, syntrophic acetate oxidising bacteria, and hydrogenotrophic *Methanothermobacter*, contributing to the enhanced methane production. Vidmar et al. [31] utilised the anaerobic bacterium *Pseudobutyrivibrio xylanivorans Mz5*^*T*^ to pretreat biomass under mesophilic and thermophilic conditions. This pretreatment targeted the hydrolysis of resistant cell wall components, such as cellulose and hemicellulose. Additionally, Costa et al. [32] used the strains *Clostridium cellulolyticum*, *Caldicellulosiruptor saccharolyticus*, and *Clostridium thermocellum* for biological co-treatment as a pretreatment for enhancing the degradation of poultry litter.

To investigate the potential of bioaugmentation by anaerobic hydrolytic bacteria, Čater et al. [21] conducted a study that included a BMP assay. Active microorganisms from a full-scale upflow anaerobic sludge blanket reactor treating brewery wastewater, along with brewery spent grain as a representative lignocellulosic substrate, were used pure and mixed cultures of *Ruminococcus flavefaciens 007C*, *Pseudobutyrivibrio xylanivorans Mz5*^*T*^, *Fibrobacter succinogenes S85*, and *Clostridium cellulovorans* were employed to enhance lignocellulose degradation and increase biogas production. *P. xylanivorans Mz5*^*T*^ exhibited the highest methane production increase (+ 17.8%), followed by specific co-cultures that also demonstrated improvements. In regard to hydrolysis and compared to the upper examples on bioaugmentation, the work from Čater et al. stands out as it highlights the possibility to combine more than one microbe on a combined approach. Fingerprinting techniques revealed significant changes in the microbial community structure, highlighting the impact of the experimental conditions on microbial dynamics. Nevertheless, Čater et al. are not the only authors, which work with multi-species bioaugmentation. Similarly to Čater et al., Senko et al. [33] employed *Clostridium acetobutylicum*, *Pseudomonas sp*., and *Enterococcus faecalis* not only for lignocellulosic waste, but also for substrates containing antibiotics and pesticides. With the combination of strains, biogas production significantly increased, demonstrating the effectiveness of immobilised anaerobic sludge cells in enhancing methanogenesis.

Apart from tackling lignocellulose degradation, there are other substrates which are difficult to degrade, such as biodiesel, which has been addressed by Sarkar et al. [34]. For this, they performed bioaugmentation with *Enterobacter*, *Pandoraea*, and *Burkholderia* strains in order to effectively biodegrade hydrocarbons. Also working with diesel, Loureiro et al. [35] has shown the suitability of *Pseudomonas aeruginosa*, *Pseudomonas stutzeri*, and *Pseudomonas mendocina*. It is further possible to lower negative impacts from contaminations, e.g., with heavy metals. In a study by Guo et al. [36], *Paracoccus* sp. was utilised in bioaugmentation experiments with plant residues rich in heavy metals, significantly enhancing degradation efficiency and boosting biogas and methane production. In the studies by Puyol et al. [37] and Tartakovsky et al. [38], bioaugmentation was tested to improve the degradation of toxic compounds in anaerobic systems. Puyol et al. used *Desulfitobacterium* strains to bioaugment the degradation of 2,4,6-trichlorophenol (246TCP), a chlorinated pesticide, but found no significant improvement in its anaerobic biodegradation. Similarly, Tartakovsky et al. [38] bioaugmented reactors with the aerobic biphenyl degrader *Rhodococcus sp. M5* for the degradation of Aroclor 1242, a PCB mixture, but observed no enhanced performance, with similar degradation rates in both bioaugmented and non-bioaugmented reactors.

So far, the majority of the studies focussed on the co-digestion of biomass in classical co-digesters. However, and apart from this, hydrolytic strains hold further the potential to improve other processes such as wastewater treatment. In this regard, a promising approach of bioaugmentation was presented for the anaerobic digestion of waste-activated sludge, where the hydrolytic bacteria, *Bacteroides uniformis* and *Clostridium sp.* were introduced at different dosages. The bioaugmentation resulted in a remarkable enhancement in methane conversion from waste-activated sludge. In another study on wastewater treatment conducted by Poszytek et al. [39, 40], the use of *Rummeliibacillus sp. POC4*, *Ochrobactrum sp. POC9*, and *Brevundimonas sp*. *LPMIX5* was investigated for enhancing hydrolysis and biogas production. The results demonstrated a significant increase of 22% and 28% in hydrolysis and biogas production, respectively, when *Rummeliibacillus sp. POC4* and *Ochrobactrum sp. POC9* were used with sewage sludge. The most significant methane yield of 298.1 mL CH_4_/g COD, with an impressive 85.2% COD conversion efficiency, was achieved when *Bacteroides uniformis* and *Clostridium sp*. were added at 100 and 900 CFU/mL, respectively [41]. In regard to wastewater treatment, another interesting article can be found by Li et al. [42]. Li et al. applied bioaugmentation to enhance the cellulose degradation capacity of an upflow anaerobic sludge blanket (UASB) reactor utilised for treating swine wastewater, a composite microbial consortium was introduced as a bioaugmentation strategy. The objective was to bolster the reactor's efficiency in breaking down cellulose, a complex organic compound found in the wastewater. The results indicated that the microbial community structure changed significantly, with the inoculated bacteria *Flavobacterium mizutaii* and *Pseudomonas* as the dominant strains in the reactor. Due to bioaugmentation, bacterial populations such as *Clostridia*, *Acidobacteria*, and *Nitrospira* were present in the bioaugmented system. Dominant groups like *Chloroflexi* and *Acidobacteria*, and several groups of *Bacteroidetes*, *Proteobacteria*, and *Firmicutes* either had much lower density or disappeared in the incubated bacterial system. By incorporating this specialised consortium of microorganisms into the reactor's microbial community, it was intended to improve the overall degradation performance and optimise the treatment process [42]. In regard to wastewater treatment it needs to be highlighted that there are cases, which can’t be attributed to any of the four phases. In this regard, a study by Lin et al. [43] can be mentioned, which used *Pseudomonas aeruginosa* for denitrification treatment processes.

A very interesting approach was shown by Lü et al. [44]. They focused on the degradation of sludge digestate through thermophilic anaerobic digestion with the addition of thermophilic, proteolytic *Coprothermobacter proteolyticus* and/or methanogenic granular sludge. The application of a proteolytic microbe is interesting, as this shows an alternative approach to the cellulolytic approaches further above. In the study by Lü et al., the sludge digestate stabilised by mesophilic anaerobic digestion was further degraded through thermophilic anaerobic digestion using 0–10% (v/v) of thermophilic, proteolytic *Coprothermobacter proteolyticus*, and/or methanogenic granular sludge. This optimisation step, conducted prior to the bioaugmentation, demonstrated that the temperature shift to thermophilic conditions promoted abiotic solubilisation of proteins and reactivated fermentative bacteria and methanogens indigenous to the sludge digestate, resulting in a final methane yield of 6.25 mmol-CH_4_/g-volatile suspended solid (VSS) digestate. The inclusion of *Coprothermobacter proteolyticus* accelerated hydrolysis and fermentation during the early stages of thermophilic anaerobic digestion and stimulated methane production through syntrophic cooperation with methanogenic granular sludge, achieving a final methane yield of 7 mmol-CH_4_/g-VSS digestate with significant protein and polysaccharide degradation.

Next to lignocellulose and refractory proteins, the successful degradation of lipids is a challenge too. In this regard, a promising approach of bioaugmentation was presented in a study conducted by Cirne et al. [45]. They used the lipolytic bacterial strain *Clostridium lundense (DSM 17049 T), which* was found to enhance methane production during anaerobic digestion of lipid-rich waste. The research demonstrated a higher methane production rate of 27.7 cm^3^ CH_4 (STP)_ g^−1^ VS _added_ day^−1^ (VS, volatile solids) under methanogenic conditions. Moreover, the bioaugmentation strategy significantly improved the hydrolysis of the lipid fraction, evident from the highest initial oleate concentration of 99% in the substrate. Xiao et al. [46] investigated lipid-rich substrates as well. In their particular case, they investigated the effects of microbial bioaugmentation on methane production in thermophilic anaerobic digestion (TAD) from food waste. Similar to Čater et al. [21], they performed bioaugmentation with an inoculum consisting of more than one strain. They used a combined inoculant of *Clostridium thermopalmarium HK1* and *Caldibacillus thermoamylovorans QK5*, which improved cumulative methane production by 24.77% compared to the control. Interestingly, the combined inoculant enriched carbohydrate- and protein-degrading bacteria, boosting carbohydrate metabolism, amino acid metabolism, and methane metabolism. This strategy specifically enhanced the methanogenesis step by promoting the tricarboxylic acid cycle (TCA cycle) and the conversion of CO_2_ to methane.

So far, all approaches focused on the improved hydrolysis of various substrates. However, and especially in the case of separate hydrolysis stages, hydrogen production is another important objective. It could be assigned to both, hydrolysis and acidogenesis. An example for this is the work by Mazzurco Miritana et al. [47], who tested bioaugmentation with a fermenting bacteria pool and anaerobic fungi (*Orpinomyces sp. and Neocallimastix sp.*) to enhance both, the “fermentative and hydrolytic phases” during anaerobic digestion of shrimp processing waste. Another example, where both phases are addresses, has been published by Zhang et al. [20]. They explored bioaugmentation with *Thermoanaerobacterium thermosaccharolyticum W16* to enhance thermophilic hydrogen production from corn stover hydrolysate. The addition of a small amount of strain *W16* (5% of total microbes) resulted in increased hydrogen yields across different seed sludge types. This bioaugmentation process also influenced the composition of soluble metabolites, favouring acetate production and reducing butyrate and ethanol accumulation in specific situations. Microbial community analysis revealed the dominance of *Thermoanaerobacterium spp.* and *Clostridium spp.* The abundance of Thermoanaerobacterium indicates that this genus was enriched successfully in the respective reactor due to bioaugmentation. It is interesting that apart from *Thermoanaerobacterium* the genus *Clostridium* was increased too, which suggests a vital role for this genus in thermophilic hydrogen generation. For some substrates, such as rotten corn stover and sludge from anaerobic digestion, the bioaugmentation significantly increased the relative abundance of strain *T. thermosaccharolyticum W16* in the microbial community, likely contributing to the enhanced hydrogen production as well. In a similar study Huang et al. [48] investigated the use of *T. thermosaccharolyticum MJ2* and biochar to enhance thermophilic hydrogen production from sugarcane bagasse. The study found that bioaugmentation with *MJ2* significantly increased hydrogen production by 95.31%, and the addition of biochar further enhanced this by an impressive 158.10%.

In addition, Camargo et al. [49] used a strain closely related to *Enterococcus casseliflavus* for hydrogen production. The strain was isolated from citrus by-products and demonstrated significant hydrogen production from xylose. Additionally, in a study by Kumar et al. [50], bioaugmentation with *Escherichia coli XL1-Blue* and *Enterobacter cloacae DSM 16657* significantly improved hydrogen production from beverage industrial wastewater. The addition of facultative anaerobic bacteria, combined with nutrients such as yeast extract and tryptone, led to a remarkable increase in hydrogen production, especially when both the bacteria and nutrients were used together. As mentioned already further above, it can be promising to use more than one strain in a combined approach. This is also possible in hydrogen production. In this regard, Laocharoen et al. [51] investigated bioaugmentation for hydrogen production by adding *Rhodobacter sphaeroides KKU-PS5* and *Lactobacillus delbrueckii ssp. bulgaricus TISTR 895* into anaerobic digesters. While the co-cultivation faced challenges due to differences in metabolic types, this study highlighted the potential of combining these strains to improve hydrogen production through bioaugmentation. In a similar approach, Sharma & Melkania, [52] evaluated the effect of bioaugmentation with three bacterial species (*Escherichia coli, Bacillus subtilis*, and *Enterobacter aerogenes*) on hydrogen production from the organic fraction of municipal solid waste. It is also possible to improve methanogenic communities due to the inoculation of hydrolytic strains. In this regard, Jung [53] investigated the impact of bioaugmentation with the mesophilic cellulose-degrading strain *Clostridium cellulolyticum H10* on anaerobic digestion of cattle manure and wastewater sludge. This strain breaks down cellulose into hydrogen, acetate, and ethanol, which enhance methanogenesis. One year later, a work from Kovács et al. followed, where they specifically aimed for the inoculation of hydrogenic bacteria to improve methanogenesis. Kovács et al. [54] investigated the roles of pure hydrogen-producing cultures of *Caldicellulosiruptor saccharolyticus* and *Enterobacter cloacae* in thermophilic and mesophilic natural biogas-producing communities, respectively. Their findings indicated that enhancing biogas production was associated with an increased abundance of hydrogen producers, with the loading rate of total organic solids playing a crucial role in maintaining an altered population balance. Promising results with *Enterobacter cloacae* have again been demonstrated by Ács et al. [55].

In another study by Morales-Martínez et al. [56], the production of hydrogen gas from pretreated agave biomass. Cellulose-degrading microorganisms obtained from bovine ruminal fluid were used to enhance H_2_ production by *Clostridium acetobutylicum*. The results demonstrated the capacity of these microorganisms to hydrolyse the pretreated agave biomass and improve hydrogen gas production, highlighting the potential of bioaugmentation in biohydrogen generation. It was difficult to assign this to a clear phase because H_2_ production relates to acidification but then it was applied in regard to hydrolysis. Collectively, these findings underscore the potential of bioaugmentation strategies in optimising anaerobic digestion processes, promoting higher yields of biogas and hydrogen, and shedding light on the microbial dynamics responsible for enhanced degradation and energy recovery from various organic waste substrates. Such insights can contribute to the development of sustainable and efficient bioenergy production techniques with significant implications for renewable energy applications.

Comparing all 33 articles, species from the following families have been successfully applied in order to improve hydrolysis with the following substrates: *Clostridiaceae* (brewery spent grain), *Thermoanaerobacteraceae* (corn stover), *Thermotogaceae* (sewage sludge), *Flavobacteriaceae* (swine wastewater), *Chlorellaceae* (microalgal biomass), *Fibrobacteraceae* (brewery spent grain) and *Dictyoglomaceae* (cattle manure). As the found strains are mostly related to a better degradation of plant derived material, the importance of lignocellulose needs to be highlighted. It is well known that lignocellulose is the most abundant renewable material on the planet. It is easily accessible and also cost-effective. However, its hydrolysis process is often difficult to complete due to its complex structure.

To move on to the next chapters, the case of Wang et al. [57] will be described at this point. They evaluated improved thermophilic anaerobic digestion (TAD) of food waste due to four thermophilic strains: *Ureibacillus suwonensis E11*, *Clostridium thermopalmarium HK1*, *Bacillus thermoamylovorans Y25*, and *Caldibacillus thermoamylovorans QK5*. Results showed that cumulative methane production improved by 2.05% (*E11*), 14.54% (*HK1*), 19.79% (*Y25*), and 9.17% (*QK5*) compared to the control. Analysis of microbial community composition revealed increased relative abundance of key hydrolytic bacteria, but also methanogenic archaea. This highlights that the impact on the microbiome cannot just be attributed to the functionality of the strains added. In the particular case of Wang et al., the addition of hydrolytic bacteria was also affecting methanogenic archaea.

### Manipulation of acidogenesis

Previous research studies have investigated the role of bioaugmentation in acidogenesis. With the chosen search terms, several articles were found, which were not only addressing anaerobic methane production, but also dark fermentation related articles. Although the present article is primarily not focussed on dark fermentation, these articles were not excluded from the present set of literature. Acidogenesis is a crucial stage responsible for converting complex organic compounds into valuable products such as volatile fatty acids (VFAs) and hydrogen gas. In recent years, microbial bioaugmentation has emerged as a promising approach to enhance acidogenesis efficiency by introducing specific microbial species or mixed cultures into anaerobic systems. This section summarises and analyses several studies that explore the impact of different microbial bioaugmentation strategies on acidogenesis performance (Table 2). It needs to be highlighted again that it is difficult to separate between hydrolytic and acidogenic bacteria, because many organisms are able to do both. To cope with this conflict, the authors have shifted articles into the acidogenesis section, if they were discussing the impact on volatile fatty acid (VFA) formation specifically. Articles were also shifted into the acidogenesis section, if an improved hydrogen formation was addressed, but without linking this specifically to syntrophic acetogenesis. Doing this, remaining studies were exclusively related to dark fermentation. In this regard, many of the found articles focus on the production of butyric acid and hydrogen gas (dark fermentation). It needs to be highlighted that production and extraction of butyric acid and/or hydrogen is something, which is usually not wanted in biogas plants, as they are supposed to enrich methane and not hydrogen or organic acids. Therefore, it is crucial to distinguish clearly which of these scenarios (dark fermentation or methane production) is addressed when investigating the impact of bioaugmentation on acidogenesis. To distinguish between hydrolysis and acidogenesis is not always simple. In this regard, Yang et al. [58] used *L. hydrogenispora ethanolica LX-B* in bioaugmentation experiments, which significantly improved hydrogen production from complex substrates. The bioaugmentation with *LX-B* resulted in hydrogen yields more than twice that of the control group in batch cultivation. Regarding dark fermentation, the improved formation of hydrogen could be subjected to the acidogenesis chapter. However, since improved degradation of complex substrates is addressed, it could also be possible to discuss this article in the hydrolysis section. Another example which fits clearer into the acidification section, has been published by Atasoy & Cetecioglu, [59]. They investigated the enhancement of butyric acid production through bioaugmentation with *Clostridium butyricum* in mixed cultures. Anaerobic sequencing batch reactors were operated under alkaline conditions and fed with dairy industry wastewater as the substrate. Bioaugmentation with *Clostridium butyricum* significantly increased butyric acid production, indicating a positive influence of this specific microbial species on acidogenesis in respect to dark fermentation.

In addition to this, Dams et al. [60] investigated the potential of bioaugmentation with *Clostridium acetobutylicum ATCC 824* for hydrogen, organic acid, and alcohol production using residual glycerol as the carbon source. Similarly to *Clostridium butyricum*, *Clostridium acetobutylicum ATCC 824* allows for the enrichment of hydrogen. Batch experiments were conducted in pure and mixed cultures, with three different sources of inocula and the experiments were conducted with mixed cultures. The work from Dams et al. is interesting, as it shows the possibility to enrich other metabolites than just hydrogen and butyric acid. Significant yields of hydrogen, but also 1,3-propanediol were achieved when *Clostridium acetobutylicum ATCC 824* was bioaugmented into the sludge from municipal wastewater with 5 g/L of glycerol. One highlight of this work was further the application of glycerol, which is regarded as a recalcitrant substrate. According to Dams et al. and with *Clostridium acetobutylicum ATCC 824* as a microbial additive, glycerol could be a promising substrate for the generation of valuable products like hydrogen and 1,3-propanediol during dark fermentation in mixed-culture approaches. Similar as Atasoy & Cetecioglu [59] or Dams et al. [60], Goud et al. [61] evaluated the possibility of bioaugmentation for the improvement of dark fermentation as well. However, they focused their work on indigenous microorganisms, which are naturally present in the environment of dark fermentation processes. They applied three acidogenic bacterial isolates belonging to the phyla *Firmicutes* and *Proteobacteria*, in order to increase hydrogen formation, but also to cope with elevated organic: for this they used the species *Bacillus subtilis*, *Pseudomonas stutzeri*, and *Lysinibacillus fusiformis*. In addition to the work from Dams et al. [60], Wang et al. [62] also demonstrated the potential of *Clostridium acetobutylicum ATCC 824* in biohydrogen production through dark fermentation but focused on using microcrystalline cellulose as the carbon source.

Some studies on dark fermentation focus more on the production of organics acids. In this regard, the work by Zheng et al. [63] can be highlighted. They investigated a biochemical strategy to enhance propionic acid production from kitchen waste acidification through bioaugmentation with *Propionibacterium acidipropionici.* Their results showed that when the inoculum of *Propionibacterium acidipropionici* comprised 30% (w/w) of the seeding sludge, propionic acid production increased by 79.57%.

Most articles that addressed dark fermentation worked with single stages process. In this regard, Liu et al. [64] stands out who addressed bioaugmentation in a more holistic approach. They investigated the impact of bioaugmentation technology on anaerobic digestion processes by directly manipulating microbial structure through bioaugmentation in a two-stage co-digestion system. In their study, different doses of *Thermoanaerobacterium thermosaccharolyticum* were introduced into the hydrogen-producing pretreatment stage. The system was operating at 55 °C. The findings revealed that the addition of *Thermoanaerobacterium thermosaccharolyticum* at 1.12 g had the most significant impact, resulting in cumulative hydrogen and methane yields of 81.54 mL/g VS and 550.98 mL/g VS, respectively. These values were 68.72% and 84.45% higher than those of the control group. Microbial analysis indicated notable changes in microbial community structure, with an increase in the relative abundance of *Thermoanaerobacterium thermosaccharolyticum* during the hydrogen production stage. This increase led to higher levels of volatile fatty acids (VFAs) and hydrogen content, suggesting a potential influence on the acidogenesis step of anaerobic digestion. Like the other acidogenesis related studies, the work from Liu et al. is addressing dark fermentation. However, and unlike the other articles, Liu et al. were the only ones, who implemented this into a system with a subsequent methanation stage.

It needs to be highlighted that bioaugmentation in acidogenesis is not always about improving biomass degradation. It can also be about the elongation of fatty acids. In this regard, a study by Zagrodnik et al. [65] can be highlighted. They used *Clostridium kluyveri (AS* + *CK)* in chain elongation processes, where it produced medium-chain fatty acids, such as caproic acid, from a mixed substrate.

### Manipulation of acetogenesis

The acetogenesis section explores various strategies to enhance acetate production within AD. Table 3 summarises bioaugmentation studies on acetogenesis. Research has focused on utilising specific microbial strains, such as *Clostridium* and *Thermoanaerobacterium* [66] to improve acetate yields. These strains have shown promise in optimising the acetogenesis phase by facilitating more efficient conversion of intermediates into acetate. As *Bacillus* species, including *Brevibacillus sp*. *KH*_*3*_ [67], *Bacillus subtilis* [68], and *B. licheniformis* [69], have also been studied for their role in stimulating hydrolytic enzymes and enhancing AD performance, their contributions are more pertinent to the hydrolysis stage and are thus discussed in the corresponding section. In the context of acetogenesis, interactions between acetogens and methanogenic archaea or hydrogen-producing bacteria have been linked to improved biogas production. In this regard, Wang et al. [70] can be highlighted. They developed a microbial consortium entitled *D83.* For this consortium they highlighted the occurrence of *Syntrophospora bryantii*, *Sedimentibacter sp*., and *Thermosyntropha bryantii*, *Methanosarcina sp*. and *Methanobacterium ferruginis*. They further explained that D83 was dominated by hydrogen-producing acetogens, which helped to enhance methane production. Bioaugmentation with *D83* doubled methane yield and rate from glucose fermentation and improved COD removal in molasses wastewater treatment. This study highlighted hydrogen-producing acetogenesis as a key step in methanogenesis, improving both acidogenesis and methanogenesis. There are cases, where such an improvement of methanogenesis is further interwoven with syntrophic relations. Syntrophic bacteria can be involved due to their role in syntrophic acetate oxidation and hydrogen turnover [20]. However, the specific role of methanogenic archaea in bioaugmentation is beyond the scope of acetogenesis and is elaborated further in the methanogenesis section. By focusing on acetogenic strains that directly contribute to acetate formation, researchers aim to optimise this critical intermediate step in AD. In a study by Huang et al. [71], the factors influencing the growth and acetate production efficiency of the *Clostridium sp. NJUST19* strain were investigated under different environmental conditions. The experimental results of digesting waste-activated sludge (WAS) with the addition of *Clostridium sp. NJUST19* showed enhanced total suspended solids (TSS) degradation and increased concentrations of VFAs. The TSS degradation rate increased to 35.3%, which was 13.4% higher than the control group. Additionally, the maximum VFAs concentration reached 4200 mg/L, indicating a significant increase of 45.8% compared to the control group. This is another example, which shows how intertwined the different phases of anaerobic digestion are. Although the title from Huang et al. refers to acetogenesis, it remains difficult to differentiate between hydrolysis, acidogenesis and acetogenesis in this specific case. It stands out that in total just one article was fitting into the “acetogenesis” chapter. On one hand, this might indicate a research gap. Amongst the detected articles, there were almost no articles addressing syntrophic butyrate- and propionate-degrading bacteria (SBOBs and SPOBs) and it might be interesting to test the suitability of such organisms for bioaugmentation. On the other hand, missing articles on bioaugmentation with a specific focus on syntrophic, acetogenic bacteria might also be explained by difficulties in culturing such bacteria. It might well be that it is just impractical to use syntrophic, acetogenic bacteria for bioaugmentation. One study that was found, has been published by Shao et al. [72]. They studied bioaugmentation to accelerate recovery in an anaerobic sequencing batch reactor, which was exposed to an organic shock load. The bioaugmented reactor, with a butyric acid-utilising culture containing *Methanobacteriales* and *Syntrophomonas*, recovered faster [52] than the non-bioaugmented reactor (110 days), by relieving feedback inhibition and boosting propionic acid degradation. Another interesting article in the regard comes from Tale et al. [73], who utilised a propionate-degrading enrichment culture dominated by *Methanospirillum hungatei* and *Methanobacterium beijingense* to bioaugment anaerobic digesters. This approach enhanced recovery after organic overload by reducing acid accumulation and shortening recovery time by approximately 25 days, demonstrating the effectiveness of bioaugmentation in improving process stability. Both studies, the one by Shao et al. and the one by Tale et al., include methanogens, which shows once again that it is not always possible to clearly distinguish published cases regarding the different phases of anaerobic digestion. Yet another case is the study by Akila and Chandra [74]. They isolated a psychrotrophic xylanolytic acetogenic strain, Clostridium sp. PXYL1, and the *Methanosarcina* strain PMET1 from a cattle manure digester. The addition of PXYL1 increased VFA levels compared to the controls, while the further addition of PMET1 enhanced biogas yields and reduced VFA levels.

An important objective regarding acidogenesis is the improved degradation of long-chain fatty acids (LCFAs). One notable study is by Cavaleiro et al. [75], which investigated the bioaugmentation of non-acclimated anaerobic granular sludge using *Syntrophomonas zehnderi* to enhance the conversion of LCFAs into methane. In this study, "non-acclimated" refers to the fact that the anaerobic granular sludge had not been previously exposed or adapted to LCFA or similar conditions before the introduction of *Syntrophomonas zehnderi.* The addition of *Syntrophomonas zehnderi* resulted in faster methane production and higher methane yields. In a similar study, Wang et al. [76] co-cultured *Syntrophomonas wolfei* and *Geobacter sulfurreducens* on the anaerobic anode of a bio-electrochemical system to degrade butyric acid. The co-culture showed a more efficient butyrate degradation than *Syntrophomonas wolfei* and methanogens. The work presented by Wang et al. stands out, as it is not just about adding a certain microbe to the process, but it combines it with the application of electrodes. With their experiment, Wang et al. indicate that the implementation of galvanic elements could be combined with bioaugmentation regarding acidogenesis.

### Manipulation of methanogenesis

The stimulation of methanogenesis through bioaugmentation primarily involves enhancing methanogenic archaea, which are crucial for converting hydrogen, carbon dioxide, and acetic acid into methane [77]. In addition, syntrophic acetate-oxidising bacteria (SAOBs) are included in this section because they play a unique role in balancing the methanogenesis pathways. Specifically, SAOBs convert acetate into hydrogen and carbon dioxide, thus bridging the gap between acetoclastic methanogenesis (which directly converts acetate to methane) and hydrogenotrophic methanogenesis (which uses hydrogen and carbon dioxide to produce methane). This balancing act helps to optimise the overall methane production in anaerobic digestion processes, making the inclusion of SAOBs in the methanogenesis section particularly relevant. Two pathways can be distinguished for methanogenesis from acetate. The first one is acetoclastic methanogenesis [78], in which acetate is enzymatically cleaved into methyl groups (converted directly to CH_4_) and carboxyl groups (oxidised to CO_2_). The second pathway involves a two-step reaction [78] performed by so-called syntrophic acetate oxidising bacteria (SAOBs). In this pathway, acetate is first oxidised by SAOBs to H_2_ and CO_2_, and subsequently, these products are further converted to CH_4_ by hydrogenotrophic methanogens.

Due to the syntrophic relationships between methanogens and bacteria, bacteria are relevant for improving methanogenesis, although they usually do not produce methane themselves. In this regard, an interesting work by Zhang et al. [10] can be highlighted. Zhang et al. was not focussing on methanogens for bioaugmentation. Instead, they were able at low ISR to accelerate methanogenesis by 78% due to the addition of *Geobacter sulfurreducens*. Fluorescence in situ hybridisation (FISH) analysis indicated a close association between *Geobacter sulfurreducens* and *methanogens*, which was attributed to syntrophic interactions between *Geobacter sulfurreducens* and methanogens affiliated with *Methanosaetaceae* and *Methanobacteriaceae*. The study from Zhang et al. [10] is of high interest due to the electrofermentative capabilities of *Geobacter sulfurreducens*. Exoelectrogenic capabilities have the potential to improve syntrophic relations between bacteria and archaea.

Similar to Zhang et al. [10], a recent work from Zhang et al. [79] focuses also on bacteria to improve methanogenesis. They used *Lactobacillus lactis* and *Bacillus velezensis* to enhance anaerobic digestion efficiency for food and kitchen waste. Both bioaugmentation and biopretreatment significantly increased crude cellulose removal rates. One might criticise that this finding belongs rather into the hydrolysis section. However, the authors describe that the observed increase in the methane yield (by 22.7–33.6%) was related to an enhanced syntrophic metabolism, which improved hydrogenotrophic methanogenesis. Once again, this shows how deeply intertwined the different phases of anaerobic digestion are. To improve syntrophic relations, it is also possible to apply conductive materials. In this regard, Xiao et al. [80] stands out, as they explored the combined application of bioaugmentation and conductive materials (CMs). Using *Clostridium pasteurianum* and CMs like biochar and magnetite, they found that hydrogenotrophic and acetoclastic methanogenesis were significantly improved.

Apart from articles focussing on improvement of syntrophic relations, there are many articles the search for way to better cope with ammonium. Ammonia has been identified as a significant inhibitor of methane production in anaerobic digestion. Numerous studies have been conducted to investigate this inhibition and develop strategies to mitigate its adverse effects. In this regard, Yang et al. [81] presented a work on bioaugmentation, where they tested seven pure strains of microorganisms to recover anaerobic digestion processes, which were suffering from ammonia inhibition. Amongst these strains are obligate acetoclastic methanogens, facultative acetoclastic methanogens, hydrogenotrophic methanogens, and as well syntrophic acetate oxidising bacteria (SAOBs). The fact that syntrophic bacteria are used to reduce the inhibitory effects of ammonium shows that the two issues are not mutually exclusive. Ammonium thus also seems to have an influence on the syntrophic interaction between bacteria and methanogenic archaea. It is worth noting that obligate acetoclastic methanogens and facultative acetoclastic methanogens are the primary groups capable of converting acetate directly to methane (HAc → CH ₄), which is why they are specifically highlighted in this section. Each of the strains were added to anaerobic digestion processes and the best results were obtained with *Methanobrevibacter smithii* (hydrogenotrophic) co-inoculated with the SAOB *Syntrophaceticus schinkii*. As a result, a 71.1% increase in methane production was observed. Bioaugmentation with *Methanosarcina barkeri* alone proved also to be efficient, enhancing both acetoclastic and hydrogenotrophic methanogenesis with a 59.7% higher methane production. That *Methanosarcina barkeri* showed good results even without the addition of SAOBs can be explained by the fact that *Methanosarcina* can also grow acetoclastically and not just hydrogenotrophically. Interestingly, a negligible improvement was achieved with *Methanothrix*, which is purely acetoclastic*.* In this regard the work by Chen et al. [82] is of high interest. They studied the recovery of anaerobic digestion systems under ammonia inhibition. The observed that especially genus *Methanosarcina* recovered fast. The recovery correlated with an increased abundance of the *Firmicutes* genera *Tissierella* and *Lutispora*. Based on these findings, one can argue that both, the hydrogenotrophic and the acetoclastic pathway is important. Yet another study, which confirms the importance of acetoclastic methanogens has been published by Jain et al. [83], who isolated *T53BJ* from the genus *Methanospirillum spp*. to enhance the biogas yield. Interestingly, Yang et al. also investigated the taxonomic profile upon bioaugmentation. The 16 s rRNA gene sequencing results showed that *Methanobacterium spp.* and *Methanothrix spp.* were the dominant archaea in all 14 reactors, regardless of the bioaugmentation. Even after inoculation of *Methanobrevibacter smithii* or *Methanosarcina barkeri*, *Methanothrix* prevailed. In fact, the relative abundances of *Methanobrevibacter smithii* or *Methanosarcina barkeri* remained below < 2%. Despite being non-dominant archaea, *Methanobrevibacter spp.* and *Methanosarcina spp.* played pivotal roles in determining the overall microbial consortium and, in turn, improved the overall performance of anaerobic digestion.

In a follow-up study, Yang et al. [77] used the same strains as mentioned above. This time, they investigated the effectiveness of the respective strains in enhancing methane (CH_4_) production during long-term approaches. The results confirmed the previous findings. Again, *Methanosarcina barkeri* or a combination of *Syntrophaceticus schinkii* and *Methanobrevibacter smithii* resulted in a remarkable increase (35%) in methane potential, although the increase was lower compared to the study before. The lowered increase in the methane potential might indicate that bioaugmentation does not allow a permanent change in the functionality of the underlying microbiome and that bioaugmentation requires a regular and continuous effort of microbial inoculation. Bottles bioaugmented with *Methanosaeta harundinacea (MSH)*, *Syntrophaceticus schinkii*, and *M. smithii* exhibited a significant increment of 49% in methane potential. These findings demonstrate the importance of enhancing both the acetoclastic and hydrogenotrophic methanogenic pathways, while underscoring the need for careful selection of bioaugmentation strains to achieve synergistic effects. Findings as the one from Yang et al. raise the question, whether the inoculation of complex consortia might be more promising than just one strain. Again, Yang et al. performed a taxonomic analysis. Unlike in the study from 2019, *Methanosarcina spp.* prevailed in the archaeal population. Results from Yang et al. are in accordance with a former study from Fotidis et al. [84]. They applied an ammonia-tolerant syntrophic acetate-oxidising (SAO) co-culture, comprising *Clostridium ultunense spp. nov.* and *Methanoculleus spp*. strain MAB1. This co-culture was tested in a mesophilic up-flow anaerobic sludge blanket (UASB) reactor under high ammonia loads [84]. Bioaugmentation of the SAO co-culture in the UASB reactor alone was not successful, likely due to the slow growth rate of the culture caused by the methanogenic partner. In contrast, when a fast-growing hydrogenotrophic methanogen, *Methanoculleus bourgensis MS2*^*T*^, was added to the SAO co-culture in fed-batch reactors, a 42% higher growth rate was observed. In accordance with Yang et al., these results show the high potential of using consortia that comprise more than just one strain. Fotidis et al. [85] also investigated methanogenic pathways and community composition in full-scale biogas digesters. They investigated these reactors under varying ammonia levels. At high ammonia concentrations, syntrophic acetate oxidation combined with hydrogenotrophic methanogenesis was dominant, with *Methanomicrobiales spp.* (in thermophilic conditions) and *Methanobacteriales spp.* (in mesophilic conditions) being the key methanogens. In contrast, low ammonia levels favoured the acetoclastic methanogenic pathway. Fotidis et al. [86] presented another study, in which ammonia inhibition was addressed. They have shown that bioaugmentation with ammonia-tolerant *Methanoculleus bourgensis MS2*^*T*^ reversed ammonia inhibition by up to 90% in a continuous stirred tank reactor. Interestingly, Fotidis et al. were not applying a pure culture. Instead, they used an enriched culture, which makes the process more practical. The counteracted ammonia toxicity increased the methane production by 36%. Sequencing revealed a shift in microbial composition, with increased bacterial diversity and reduced archaeal diversity in the bioaugmented reactor. Ammonia-tolerant methanogens dominated, outperforming pure cultures by 25%. Similarly to Fotidis et al., Tian et al. [1] investigated the importance of *M. bourgensis* regarding ammonia inhibition too. They observed that bioaugmentation of *M. bourgensis* under extreme ammonia conditions (11 g NH_4_^+^-N L^−1^) resulted in an immediate increase of 28% in methane production. The fact that multiple studies highlighted the possibility to overcome ammonia inhibition due to addition of *M. bourgensis*, underscores the importance of this species.

In another study on ammonia inhibition, Yan et al. [87] assessed the application of bioaugmentation with *Methanoculleus sp. DTU887*. Due to the bioaugmentation, a digester fed with the organic fraction of municipal solid waste showed an increase in the methane yield of 21%. Next to the biogas productivity, Yan et al. were also assessing the concentration of VFAs. They observed a reduction of 10% in VFAs compared to the pre-bioaugmentation period, which indicates a more efficient VFA consumption. Two years later, Yan et al. [88] continued their research on ammonia-tolerant methanogens. They explored a novel bioaugmentation method, combining gel-immobilised ammonia-tolerant methanogens (biogel) with biochar, to alleviate ammonia inhibition in thermophilic anaerobic systems. Four reactors were subjected to ammonia shocks: one with biogel, one with biochar, one with both, and a control. Results showed that reactors receiving both supplements achieved 100% methane production recovery, while the other configurations showed methane production losses. They described further that reaction with biochar, or the combination of biochar and biogel facilitated the adaptation to higher ammonia levels. It attracts attention that multiple studies tackled ammonia inhibition successful by applying *Methanoculleus* or consortia containing *Methanoculleus* [84, 86, 89]. In yet another study, Fotidis et al. [90] revealed that an increase in methane levels in ammonia-rich environments could be directly linked to the presence of *Methanoculleus*. They introduced a fast-growing hydrogenotrophic methanogen, *Methanoculleus bourgensis MS2*^*T*^, into a reactor with high ammonia levels, achieving a 31.3% increase in methane production. High-throughput gene sequencing showed a fivefold rise in *Methanoculleus* spp. abundance after bioaugmentation. Although these results on *Methanoculleus* appear quite promising, there are other hydrogenotrophic methanogens, which could also help to overcome ammonia inhibition. In this regard, Wang et al. [91] worked with four different hydrogenotrophic methanogens, namely *Methanoculleus bourgensis*, *Methanobacterium congolense, Methanoculleus thermophilus*, and *Methanothermobacter thermautotrophicus.* These hydrogenotrophic methanogens were applied together with two SAOBs, *namely Tepidanaerobacter acetatoxydans* and *Thermacetogenium phaeum*. Under different ammonia concentrations (0.26, 3, 5, and 7 g NH_4_^+^-N L^−1^), all strains showed the potential to improve the process performance. Yet in another study, Gállego-Bravo et al. [92] studied enhanced methane production from municipal waste by bioaugmenting a thermophilic anaerobic digestion process with a hydrogenotrophic methanogenic community. Interestingly, they did not work on ammonia inhibition. Instead, the bioaugmentation improved methane yield from the organic fraction of municipal solid waste by 4%. This indicates that bioaugmenting anaerobic digesters with hydrogenotrophic methanogens is useful for more scenarios than just ammonia inhibition. Key microbes involved were the archaeon *Methanoculleus* and bacterial order *MBA08*.

To overcome ammonia inhibition the application of oxygen might be interesting too. Although not linked to ammonia inhibition, there is a work the combines the application of oxygen and bioaugmentation. Hua et al. [93] explored the use of micro-aerobic microbial communities at elevated temperatures. By introducing the methanogens *Methanosarcina acetivorans C2A* and *Methanosaeta thermophila NBRC* 101360, they achieved a significant increase in biogas production of about 44.78%.

So far, most articles about methanogenesis addressed ammonia inhibition or improved syntrophic relations. But there are other stressors that could impair methanogenic communities and, in this regard, it could be interesting to apply methanogens, which can better cope with acidosis (Table 4). That this is possible was already mentioned earlier with the work by Chen et al., who highlighted the fast recovery of *Methanosarcina*. Another interesting work to cope with acidosis has been presented by Savant et al. [94]. They used the acid-tolerant hydrogenotrophic methanogen *Methanobrevibacter acididurans* to enhance methane production and reduce VFA accumulation in acidic anaerobic digesters. In another study, Li et al. [4] demonstrated that *Methanosaeta* dominated in anaerobic digestion in oxytetracycline contaminated sludges under acidic conditions with a pH of 4.6 at the first compartment of an anaerobic baffled reactor. In a further study by Town & Dumonceaux [95], they introduced an acetoclastic consortium into acidified batch digesters, which significantly reduced acetate accumulation and increased methane production. PCR analysis revealed a substantial increase in an acetoclastic methanogens related to *Methanosarcina sp*, which highlights once again the high potential of the genus *Methanosarcina*.

### Extraordinary approaches in bioaugmentation research

In Tables 1, 2, 3 and 4 the use of controls has been assessed as described further above. Numbers 1–4 were used to indicate whether controls were applied. However, not in all experimental setups it is possible or useful to have control experiments, where no cells, autoclaved or non-autoclaved cells are added. Several experiments are not focused on biogas production. Some of them have an unusual set-up, which is designed to evaluate selected strains rather than a complex microbiome. In Tables 1, 2, 3 and 4 such extraordinary cases have been defined as “other”. This concerns for example the case of Arkatkar et al. [19]. The authors assessed coculture conditions for multiple strains based on redox activity, electron transfer rate, columbic efficiency, and internal resistances in a microbial fuel cell, which is very different from typical anaerobic digestion experiments. In such experiments, the aim is not to implement a certain strain into a complex microbiome and therefore, no controls are needed. At least not in the sense as it was analysed in Tables 1, 2, 3 and 4. Nevertheless, such experiments have a certain importance for bioaugmentation. Mapping of microbial interactions can help to define conditions, which might be relevant for bioaugmentation in practice. Another exotic example is from Rinland & Gómez, [96], who have searched for strains that allow better degradation of onion waste. In this case, multiple strains were isolated from onion waste. However, Rinland and Gómez did not work with complete methanogenic communities. They selected strains that showed good degradation capabilities, and in this case, biogas formation was not a suited criterion to evaluate the experimental success. The authors isolated strains from onion waste at different degradation stages and locations. Growth patterns and carbon source utilisation of the isolates were analysed to identify promising candidates. Among the selected strains, *Bacillus subtilis sp.MB2-62* and *Pseudomonas poae VE-74* demonstrated characteristics making them potential candidates for bioaugmentation or pretreatment in anaerobic digestion processes. As control, they always used a sterilised tube without any active microbes. The work from Rinland & Gómez was taken into account as they were screening for microbes with potential for bioaugmentation, although these strains were not applied in bioaugmentation experiments yet.

Amongst the works, which compared different strains in regard to their biogas formation potential, Jones et al. [97] was also found as an extraordinary example. They focused on methane generation from nonproductive coal. Coal is a rather unusual substrate for biogas formation, which usually is not degraded. However, the respective production sites contain degradable coal intermediates (geopolymers), for which Jones et al. were highlighting their potential in respect to biogas formation as a potential fuel source. The researchers stimulated methane production using two approaches: biostimulation with nutrient supplementation and bioaugmentation with a consortium of bacteria and methanogens enriched from wetland sediment. The approach differs strongly from typical anaerobic digestion approaches, as they were not starting the experiments with manure from animals or water treatment, which is usually the case in the biogas industry. Apparently, the coal had some intrinsic methanogenic activity, which can be stimulated with nutrients. The biogas formation was even better, if they used a mixed culture from wet-lands. However they did not describe any control, where autoclaved cells were added. So it is difficult to say, which amount of biogas could be attributed to the amount of COD, which was present in the cell mixture added.

Although the focus of the present work was not on microbial fuels cells (MFCs), the used search terms also related to some articles, which used this technology. One work that can be highlighted here is from Arkatkar et al. [19]. They worked with pure cultures, which is usually not regarded as bioaugmentation. However, Arkatkar et al. analysed the coculture behaviour of multiple species. A deeper understanding on how different strains interact and how they might be combined is indeed of interest for better understanding of bioaugmentation. Arkatkar et al. performed coculturing experiments in the anodic chamber of multiple strains, namely *Pseudomonas aeruginosa BR*, *Alcaligenes faecalis SW* and *Escherichia coli EC*. Arkatkar highlighted that coculturing *Pseudomonas aeruginosa BR* with *Alcaligenes faecalis SW* or *Escherichia coli EC* improved the energy generation in both cases. Although the primary goal for Arkatkar et al. was to improve the performance of MFCs and not typical digesters, such experiments can help to define synergies between microorganisms. Although not found with the search terms applied in the systematic search for this study, similar works can be found, when specifically searching for this. For example, a recent work has demonstrated a light driven carbon dioxide reduction to methane by *Methanosarcina barkeri* in an electric syntrophic coculture [98]. Bagchi & Behera [99] investigated the impact of bioaugmentation on microbial fuel cells (MFCs) by introducing *Pseudomonas aeruginosa* into anaerobic sludge (MFC_P_) and comparing its performance to a control MFC seeded with mixed anaerobic sludge (MFC_C_). They also tested an additional MFC with intermittent aeration and bioaugmentation (MFC_P+A_). The results showed that MFC_P+A_ produced significantly more electricity than MFC_P_ alone, with a 4% increase compared to MFC_P_ and a 31% increase compared to MFC_C_. This improvement was due to better organic degradation and more efficient electron transfer in the bioaugmented MFCs. The MFC_P+A_ configuration achieved a coulombic efficiency of 10.4%, which was higher than both MFC_P_ (9.7%) and MFC_C_ (4.41%). These findings are relevant to anaerobic digestion because they demonstrate that bioaugmentation with specific microorganisms, along with intermittent aeration, can enhance the efficiency of electron transfer and electricity generation. This approach can potentially be applied to improve anaerobic digestion processes by optimising microbial activity and enhancing overall system performance.

Finally and in regard to extraordinary approaches, wastewater treatment should be highlighted. Lin et al. [43] used *Pseudomonas aeruginosa* for denitrification treatment processes. This process is difficult to relate to any of the four phases of anaerobic digestion.

## Conclusions

In conclusion, the literature underscores the significant potential of augmenting microorganism populations to amplify biomethane production within anaerobic digestion (AD) systems. Bioaugmentation offers the potential to improve yield, speed and robustness through increased biomass conversion, faster digestion rates and/or enhanced process stability. While much of the research has been confined to laboratory settings, the prospect of scaling-up these strategies appears promising. In addition to the potential improvements that bioaugmentation brings to the AD process, several other aspects must be studied before new bioaugmentation related products or technologies can be introduced on the market. These include scale-up studies (including repeated additions and long-term performance monitoring versus a reference), proof of economic viability, quality and stability of the bioaugmentation product, transport, storage and handling requirements, biosafety and regulatory aspects.

The focus of bioaugmentation primarily on the hydrolysis/acidogenesis phase of anaerobic digestion (AD) is logical, as this stage plays a critical role in enhancing the degradation rate and yield of lignocellulosic compounds. This is particularly important given the abundance of lignocellulosic waste, which serves as the primary feedstock for AD and is often subjected to various stressors. The studies reviewed include a variety of microbial populations, ranging from single species to simple and complex consortia. Notably, multiple articles have shown that the augmentation of just one or a few species can significantly impact the composition of the entire microbiome, underscoring the importance of a metataxonomic approach to study the dynamics of bioaugmentation. Best practice recommended by the authors (Table 5) is to not only map the bioaugmentation culture itself and the microbiome of the anaerobic digester inoculum before bioaugmentation, but also monitor the anaerobic digester after bioaugmentation. This approach helps in understanding how bioaugmentation influences the microbial community structure and functionality over time, which is crucial for optimising AD processes. Alongside taxonomic screening we recommend putting more attention on conducting the research at a recommended ISR to ensure practical relevance of the results. Moreover, research has consistently demonstrated that the addition of co-cultures or small consortia often produces more significant effects compared to the augmentation of single species. This highlights the potential of mixed-culture bioaugmentation as a promising field for further study, as these consortia can better mimic natural microbial communities, leading to more robust and efficient degradation processes. Among the microorganisms studied, numerous efficient species, such as those from the *Clostridiaceae* family, have shown particular promise in enhancing the hydrolysis/acidogenesis phase. Hydrogenotrophic methanogenic archaea have a great potential in improving digester robustness, it stands out that *Methanoculleus* was used most often. Additionally, despite the few articles, *Methanosarcina* methanogens exhibit remarkable resilience and versatility in biomethane production, making them prime candidates for enhancing the methanogenesis phase and avoiding the accumulation of acetate or hydrogen in digester systems. This improved speed and robustness is opening up the possibility of increasing digester OLR. Despite their potential, research into their utilisation in bioaugmentation remains limited. One reason for this might be the difficulties in culturing them in pure culture. Leveraging these archaea could yield substantial benefits, particularly in addressing volatile fatty acid accumulation during the hydrolysis/acidogenesis phase. Thus, further exploration and implementation of bioaugmentation strategies, especially involving mixed cultures and key species like *Methanosarcina*, hold great promise for optimising AD processes and advancing sustainable biogas production.

**Table 5:** Checklist for effective bioaugmentation in anaerobic digestion

Category	Checklist items
General preparation	• Use ≥ 3 technical replicates • Clearly define inoculum origin (e.g., digested sewage sludge, co-digester sludge) • Ensure inoculum is adapted to the feedstock used • Verify sufficient microbial activity (e.g., ISR or SMA) • Standardise reactor setup (batch volume, headspace, mixing, temperature regime) • Document all process parameters: temperature, TS, VS, C:N ratio, NH₄⁺-N/NH₃, pH, VFAs, alkalinity, conductivity, biogas volume, biogas composition, feedstock characteristics • Indicate the anaerobic digestion stage intended for optimisation (hydrolysis, acidogenesis, acetogenesis, or methanogenesis)
Controls to avoid false positives/negatives	• Negative control: no bioaugmentation • Negative control: addition of autoclaved strains • Negative control: strain known to show no positive effect • Positive control: strain known to produce a positive effect • Validate inoculum quality using a control substrate with known expected biogas/methane yield • Use ≥ 3 technical replicates
Microbial monitoring	• Meta-taxonomic start-point analysis (bacteria and archaea) of inoculum and augmentation culture as well as the feedstock mix • Meta-taxonomic end-point analysis • Meta-taxonomic time-series monitoring and/or end-point analysis to detect community shifts • Determine the COD contributed by the added microorganisms and calculate the expected biogas/methane yield that could be produced from this COD

## Data Availability

No datasets were generated or analysed during the current study.

## Abbreviations

AD: Anaerobic digestion
SLR: Systematic literature review
SPO: Syntrophic propionate oxidation
SBO: Syntrophic butyrate oxidation
CH₄: Methane
OLR: Organic loading rate
BMP: Biochemical methane potential
ISR: Inoculum-to-substrate ratio
MFC: Microbial fuel cell
SE: Steam explosion
UASB: Upflow anaerobic sludge blanket
VSS: Volatile suspended solid
VS: Volatile solids
TAD: Thermophilic anaerobic digestion
TCA cycle: Tricarboxylic acid cycle
VFAs: Volatile fatty acids
WAS: Waste-activated sludge
TSS: Total Suspended Solids
LCFAs: Long-chain fatty acids

## References

[1] Tian H, Mancini E, Treu L, Angelidaki I, Fotidis IA. Bioaugmentation strategy for overcoming ammonia inhibition during biomethanation of a protein-rich substrate. Chemosphere. 2019;231:415–22. 10.1016/j.chemosphere.2019.05.140. 31146133 10.1016/j.chemosphere.2019.05.140

[2] Liu Z, Feng F, Li Y, Sun Y, Tagawa K. A corncob biochar-based superhydrophobic photothermal coating with micro-nano-porous rough-structure for ice-phobic properties. Surf Coat Technol. 2023;457:129299.

[3] Im S, Petersen SO, Lee D, Kim DH. Effects of storage temperature on CH4 emissions from cattle manure and subsequent biogas production potential. Waste Manag. 2020;101:35–43. 31586875 10.1016/j.wasman.2019.09.036

[4] Li C, Wang R, Yang X, Zhou M, Pan X, Cai G, et al. Deeper investigation on methane generation from synthetic wastewater containing oxytetracycline in a scale up acidic anaerobic baffled reactor. Bioresour Technol. 2021;333:125156. 33906019 10.1016/j.biortech.2021.125156

[5] Xu X, Yan M, Sun Y, Li Y. Bioaugmentation with cold-tolerant methanogenic culture to boost methane production from anaerobic co-digestion of cattle manure and corn straw at 20℃. Chem Eng J. 2023;466:143183.

[6] Jain S, Jain S, Wolf IT, Lee J, Tong YW. A comprehensive review on operating parameters and different pretreatment methodologies for anaerobic digestion of municipal solid waste. Renew Sustain Energy Rev. 2015;52:142–54.

[7] Nzila A, Razzak SA, Zhu J. Bioaugmentation: an emerging strategy of industrial wastewater treatment for reuse and discharge. Int J Environ Res Public Health. 2016;13(9):846. 27571089 10.3390/ijerph13090846 PMC5036679

[8] Semrany S, Favier L, Djelal H, Taha S, Amrane A. Bioaugmentation: possible solution in the treatment of bio-refractory organic compounds (Bio-ROCs). Biochem Eng J. 2012;69:75–86.

[9] Tyagi M, da Fonseca MMR, de Carvalho CCCR. Bioaugmentation and biostimulation strategies to improve the effectiveness of bioremediation processes. Biodegradation. 2011;22:231–41. 20680666 10.1007/s10532-010-9394-4

[10] Zhang S, Chang J, Liu W, Pan Y, Cui K, Chen X, et al. A novel bioaugmentation strategy to accelerate methanogenesis via adding *Geobacter sulfurreducens* PCA in anaerobic digestion system. Sci Total Environ. 2018;642:322–6. 10.1016/j.scitotenv.2018.06.043. 29906723 10.1016/j.scitotenv.2018.06.043

[11] Mengist W, Soromessa T, Legese G. Method for conducting systematic literature review and meta-analysis for environmental science research. MethodsX. 2020;7:100777. 10.1016/j.mex.2019.100777. 31993339 10.1016/j.mex.2019.100777 PMC6974768

[12] Kirchherr J. Bullshit in the sustainability and transitions literature: a provocation. Circ Econ Sustain. 2023;3(1):167–72. 10.1007/s43615-022-00175-9.

[13] Angelidaki I, Alves M, Bolzonella D, Borzacconi L, Campos JL, Guwy AJ, et al. Defining the biomethane potential (BMP) of solid organic wastes and energy crops: a proposed protocol for batch assays. Water Sci Technol. 2009;59(5):927–34. 19273891 10.2166/wst.2009.040

[14] Alzate ME, Muñoz R, Rogalla F, Fdz-Polanco F, Pérez-Elvira SI. Biochemical methane potential of microalgae: influence of substrate to inoculum ratio, biomass concentration and pretreatment. Bioresour Technol. 2012;123:488–94. 22940359 10.1016/j.biortech.2012.06.113

[15] Raposo F, Borja R, Martín MA, Martín A, la De Rubia MA, Rincón B. Influence of inoculum–substrate ratio on the anaerobic digestion of sunflower oil cake in batch mode: process stability and kinetic evaluation. Chem Eng J. 2009;149(1–3):70–7.

[16] Maya-Altamira L, Baun A, Angelidaki I, Schmidt JE. Influence of wastewater characteristics on methane potential in food-processing industry wastewaters. Water Res. 2008;42(8–9):2195–203. 18191984 10.1016/j.watres.2007.11.033

[17] Valentin MT, Ciolkosz D, Białowiec A. Influence of inoculum-to-substrate ratio on biomethane production via anaerobic digestion of biomass. Environ Microbiol Rep. 2024;16(6):e70009. 39621533 10.1111/1758-2229.70009 PMC11610627

[18] Holliger C, Alves M, Andrade D, Angelidaki I, Astals S, Baier U, et al. Towards a standardization of biomethane potential tests. Water Sci Technol. 2016;74(11):2515–22. 27973356 10.2166/wst.2016.336

[19] Arkatkar A, Mungray AK, Sharma P. Bioelectrochemical behaviour of a sequentially added biocatalytic coculture in a microbial fuel cell. J Basic Microbiol. 2020;60(7):562–73. 32311138 10.1002/jobm.202000042

[20] Zhang K, Cao GL, Ren NQ. Bioaugmentation with *Thermoanaerobacterium thermosaccharolyticum* W16 to enhance thermophilic hydrogen production using corn stover hydrolysate. Int J Hydrogen Energy. 2019;44(12):5821–9. 10.1016/j.ijhydene.2019.01.045.

[21] Čater M, Fanedl L, Malovrh Š, Marinšek Logar R. Biogas production from brewery spent grain enhanced by bioaugmentation with hydrolytic anaerobic bacteria. Bioresour Technol. 2015;186:261–9. 10.1016/j.biortech.2015.03.029. 25836034 10.1016/j.biortech.2015.03.029

[22] Nielsen HB, Mladenovska Z, Ahring BK. Bioaugmentation of a two-stage thermophilic (68°C/55°C) anaerobic digestion concept for improvement of the methane yield from cattle manure. Biotechnol Bioeng. 2007;97(6):1638–43. 10.1002/bit.21342. 17252605 10.1002/bit.21342

[23] Barua VB, Goud VV, Kalamdhad AS. Microbial pretreatment of water hyacinth for enhanced hydrolysis followed by biogas production. Renew Energy. 2018;126:21–9. 10.1016/j.renene.2018.03.028.

[24] Peng X, Börner RA, Nges IA, Liu J. Impact of bioaugmentation on biochemical methane potential for wheat straw with addition of *Clostridium cellulolyticum*. Bioresour Technol. 2014;152:567–71. 10.1016/j.biortech.2013.11.067. 24355075 10.1016/j.biortech.2013.11.067

[25] Ecem Öner B, Akyol Ç, Bozan M, Ince O, Aydin S, Ince B. Bioaugmentation with *Clostridium thermocellum* to enhance the anaerobic biodegradation of lignocellulosic agricultural residues. Bioresour Technol. 2018;249:620–5. 10.1016/j.biortech.2017.10.040. 29091846 10.1016/j.biortech.2017.10.040

[26] Ozbayram EG, Kleinsteuber S, Nikolausz M, Ince B, Ince O. Enrichment of lignocellulose-degrading microbial communities from natural and engineered methanogenic environments. Appl Microbiol Biotechnol. 2018;102:1035–43. 29151162 10.1007/s00253-017-8632-7

[27] Sinha D, Banerjee S, Mandal S, Basu A, Banerjee A, Balachandran S, et al. Enhanced biogas production from *Lantana camara* via bioaugmentation of cellulolytic bacteria. Bioresour Technol. 2021;340:125652. 34332446 10.1016/j.biortech.2021.125652

[28] Shanmugam S, Sun C, Chen Z, Wu YR. Enhanced bioconversion of hemicellulosic biomass by microbial consortium for biobutanol production with bioaugmentation strategy. Bioresour Technol. 2019;279:149–55. 30716607 10.1016/j.biortech.2019.01.121

[29] Kavitha S, Subbulakshmi P, Banu JR, Gobi M, Yeom IT. Enhancement of biogas production from microalgal biomass through cellulolytic bacterial pretreatment. Bioresour Technol. 2017;233:34–43. 28258994 10.1016/j.biortech.2017.02.081

[30] Mulat DG, Huerta SG, Kalyani D, Horn SJ. Enhancing methane production from lignocellulosic biomass by combined steam-explosion pretreatment and bioaugmentation with cellulolytic bacterium *Caldicellulosiruptor bescii*. Biotechnol Biofuels. 2018;11:19. 10.1186/s13068-018-1025-z. 29422947 10.1186/s13068-018-1025-z PMC5787918

[31] Vidmar B, Logar RM, Panjičko M, Fanedl L. Influence of thermal and bacterial pretreatment of microalgae on biogas production in mesophilic and thermophilic conditions. Acta Chim Slov. 2017;64(1):25–32. 10.17344/acsi.2016.3095 28380240

[32] Costa JC, Barbosa SG, Alves MM, Sousa DZ. Thermochemical pre- and biological co-treatments to improve hydrolysis and methane production from poultry litter. Bioresour Technol. 2012;111:141–7. 10.1016/j.biortech.2012.02.047. 22391589 10.1016/j.biortech.2012.02.047

[33] Senko O, Gladchenko M, Maslova O, Efremenko E. Long-term storage and use of artificially immobilized anaerobic sludge as a powerful biocatalyst for conversion of various wastes including those containing xenobiotics to biogas. Catalysts. 2019;9(4):326.

[34] Sarkar P, Roy A, Pal S, Mohapatra B, Kazy SK, Maiti MK, et al. Enrichment and characterization of hydrocarbon-degrading bacteria from petroleum refinery waste as potent bioaugmentation agent for in situ bioremediation. Bioresour Technol. 2017;242:15–27. 10.1016/j.biortech.2017.05.010. 28533069 10.1016/j.biortech.2017.05.010

[35] Loureiro DB, Olivera C, Tondo ML, Herrero MS, Salvatierra LM, Pérez LM. Microbial characterization of a facultative residual sludge obtained from a biogas plant with ability to degrade commercial B10 diesel oil. Ecol Eng. 2020;144:105710.

[36] Guo Q, Ji J, Ling Z, Zhang K, Xu R, Leng X, et al. Bioaugmentation improves the anaerobic co-digestion of cadmium-containing plant residues and cow manure. Environ Pollut. 2021;289:117885. 10.1016/j.envpol.2021.117885. 34388552 10.1016/j.envpol.2021.117885

[37] Puyol D, Mohedano AF, Rodriguez JJ, Sanz JL. Effect of 2,4,6-trichlorophenol on the microbial activity of adapted anaerobic granular sludge bioaugmented with *Desulfitobacterium* strains. N Biotechnol. 2011;29(1):79–89. 10.1016/j.nbt.2011.06.011. 21718810 10.1016/j.nbt.2011.06.011

[38] Tartakovsky B, Michotte A, Cadieux JCA, Lau PCK, Hawari J, Guiot SR. Degradation of Aroclor 1242 in a single-stage coupled anaerobic/aerobic bioreactor. Water Res. 2001;35(18):4323–30. 11763034 10.1016/s0043-1354(01)00175-0

[39] Poszytek K, Karczewska-Golec J, Ciok A, Decewicz P, Dziurzynski M, Gorecki A, et al. Genome-guided characterization of *Ochrobactrum* sp. POC9 enhancing sewage sludge utilization—biotechnological potential and biosafety considerations. Int J Environ Res Public Health. 2018;15(7):1501. 10.3390/ijerph15071501. 30013002 10.3390/ijerph15071501 PMC6069005

[40] Poszytek K, Karczewska-Golec J, Dziurzynski M, Stepkowska-Kowalska O, Gorecki A, Decewicz P, et al. Genome-wide and functional view of proteolytic and lipolytic bacteria for efficient biogas production through enhanced sewage sludge hydrolysis. Molecules. 2019;24(14):2624. 10.3390/molecules24142624. 31323902 10.3390/molecules24142624 PMC6680700

[41] Cayetano RDA, Park J, Kim GB, Jung JH, Kim SH. Enhanced anaerobic digestion of waste-activated sludge via bioaugmentation strategy—phylogenetic investigation of communities by reconstruction of unobserved states (PICRUSt2) analysis through hydrolytic enzymes and possible linkage to system performance. Bioresour Technol. 2021;332:125014. 33839513 10.1016/j.biortech.2021.125014

[42] Li P, Wang Y, Wang Y, Jiang Z, Tong L. Bioaugmentation of cellulose degradation in swine wastewater treatment with a composite microbial consortium. Fresenius Environ Bull. 2010;19(12b):3107–12.

[43] Lin Z, Sun D, Dang Y, Holmes DE. Significant enhancement of nitrous oxide energy yields from wastewater achieved by bioaugmentation with a recombinant strain of *Pseudomonas aeruginosa*. Sci Rep. 2018;8:11916. 30093706 10.1038/s41598-018-30326-8 PMC6085377

[44] Lü F, Li T, Wang T, Shao L, He P. Improvement of sludge digestate biodegradability by thermophilic bioaugmentation. Appl Microbiol Biotechnol. 2014;98:969–77. 23681590 10.1007/s00253-013-4977-8

[45] Cirne DG, Björnsson L, Alves M, Mattiasson B. Effects of bioaugmentation by an anaerobic lipolytic bacterium on anaerobic digestion of lipid-rich waste. J Chem Technol Biotechnol. 2006;81(11):1745–52.

[46] Xiao M, Wang N, Zhang S, Hu P, Xie B, Shi J, et al. Synergistic bioaugmentation with *Clostridium thermopalmarium* and *Caldibacillus thermoamylovorans* improved methane production from thermophilic anaerobic digestion of food waste. Chem Eng J. 2024;480:148372.

[47] Mazzurco Miritana V, Gaetani A, Signorini A, Marone A, Massini G. Bioaugmentation strategies for enhancing methane production from shrimp processing waste through anaerobic digestion. Fermentation. 2023;9(4):401.

[48] Huang JR, Chen X, Hu BB, Cheng JR, Zhu MJ. Bioaugmentation combined with biochar to enhance thermophilic hydrogen production from sugarcane bagasse. Bioresour Technol. 2022;348:126790. 10.1016/j.biortech.2022.126790. 35104653 10.1016/j.biortech.2022.126790

[49] Camargo FP, Sakamoto IK, Silva EL, Duarte ICS, Varesche MBA. Bioaugmentation with *Enterococcus casseliflavus*: a hydrogen-producing strain isolated from Citrus peel waste. Waste Biomass Valorization. 2021;12:895–911.

[50] Kumar G, Bakonyi P, Sivagurunathan P, Kim SH, Nemestóthy N, Bélafi-Bakó K, et al. Enhanced biohydrogen production from beverage industrial wastewater using external nitrogen sources and bioaugmentation with facultative anaerobic strains. J Biosci Bioeng. 2015;120(2):155–60. 10.1016/j.jbiosc.2014.12.011. 25661265 10.1016/j.jbiosc.2014.12.011

[51] Laocharoen S, Reungsang A, Plangklang P. Bioaugmentation of *Lactobacillus delbrueckii* ssp. *bulgaricus* TISTR 895 to enhance bio-hydrogen production of *Rhodobacter sphaeroides* KKU-PS5. Biotechnol Biofuels. 2015;8:1–16. 26613000 10.1186/s13068-015-0375-z PMC4660636

[52] Sharma P, Melkania U. Effect of bioaugmentation on hydrogen production from organic fraction of municipal solid waste. Int J Hydrogen Energy. 2018;43(15):7290–8. 10.1016/j.ijhydene.2018.03.031.

[53] Jung S. Enhanced methane production in mesophilic anaerobic digesters added with *Clostridium cellulolyticum* H10 using cattle manure or wastewater sludge as substrates. J Korean Soc Water Sci Technol. 2012;20:13–20.

[54] Kovács KL, Ács N, Kovács E, Wirth R, Rákhely G, Strang O, et al. Improvement of biogas production by bioaugmentation. BioMed Res Int. 2013;2013:482653. 10.1155/2013/482653. 23484123 10.1155/2013/482653 PMC3591239

[55] Ács N, Bagi Z, Rákhely G, Minárovics J, Nagy K, Kovács KL. Bioaugmentation of biogas production by a hydrogen-producing bacterium. Bioresour Technol. 2015;186:286–93. 10.1016/j.biortech.2015.02.098. 25836037 10.1016/j.biortech.2015.02.098

[56] Morales-Martínez TK, Medina-Morales MA, Ortíz-Cruz AL, la Rodríguez-De Garza JA, Moreno-Dávila M, López-Badillo CM, et al. Consolidated bioprocessing of hydrogen production from agave biomass by *Clostridium acetobutylicum* and bovine ruminal fluid. Int J Hydrogen Energy. 2020;45(26):13707–16. 10.1016/j.ijhydene.2019.11.089.

[57] Wang N, Xiao M, Zhang S, Chen X, Shi J, Fu S, et al. Evaluating the potential of different bioaugmented strains to enhance methane production during thermophilic anaerobic digestion of food waste. Environ Res. 2024;245:118031. 38157970 10.1016/j.envres.2023.118031

[58] Yang Z, Guo R, Shi X, He S, Wang L, Dai M, et al. Bioaugmentation of *Hydrogenispora ethanolica* LX-B affects hydrogen production through altering indigenous bacterial community structure. Bioresour Technol. 2016;211:319–26. 10.1016/j.biortech.2016.03.097. 27023388 10.1016/j.biortech.2016.03.097

[59] Atasoy M, Cetecioglu Z. Butyric acid dominant volatile fatty acids production: bio-augmentation of mixed culture fermentation by *Clostridium butyricum*. J Environ Chem Eng. 2020;8(6):104496. 10.1016/j.jece.2020.104496.

[60] Dams RI, Guilherme AA, Vale MS, Nunes VF, Leitão RC, Santaella ST. Fermentation of residual glycerol by *Clostridium acetobutylicum* ATCC 824 in pure and mixed cultures. Environ Technol. 2016;37(23):2984–92. 27230401 10.1080/09593330.2016.1173114

[61] Goud RK, Sarkar O, Chiranjeevi P, Venkata Mohan S. Bioaugmentation of potent acidogenic isolates: a strategy for enhancing biohydrogen production at elevated organic load. Bioresour Technol. 2014;165:223–32. 10.1016/j.biortech.2014.03.049. 24751375 10.1016/j.biortech.2014.03.049

[62] Wang A, Ren N, Shi Y, Lee DJ. Bioaugmented hydrogen production from microcrystalline cellulose using co-culture—*Clostridium acetobutylicum* X9 and *Ethanoigenens harbinense* B49. Int J Hydrogen Energy. 2008;33(2):912–7.

[63] Zheng Y, Wang P, Yang X, Zhao L, Ren L, Li J. Metagenomics insight into bioaugmentation mechanism of *Propionibacterium acidipropionici* during anaerobic acidification of kitchen waste. Bioresour Technol. 2022;362:127843. 10.1016/j.biortech.2022.127843. 36031136 10.1016/j.biortech.2022.127843

[64] Liu X, Zhu X, Yellezuome D, Liu R, Liu X, Sun C, et al. Effects of adding *Thermoanaerobacterium thermosaccharolyticum* in the hydrogen production stage of a two-stage anaerobic digestion system on hydrogen-methane production and microbial communities. Fuel. 2023;342:127831.

[65] Zagrodnik R, Duber A, Łężyk M, Oleskowicz-Popiel P. Enrichment versus bioaugmentation—microbiological production of caproate from mixed carbon sources by mixed bacterial culture and *Clostridium kluyveri*. Environ Sci Technol. 2020;54(9):5864–73. 10.1021/acs.est.9b07651. 32267683 10.1021/acs.est.9b07651 PMC7588035

[66] Kuribayashi K, Kobayashi Y, Yokoyama K, Fujii K. Digested sludge-degrading and hydrogen-producing bacterial floras and their potential for biohydrogen production. Int Biodeterior Biodegradation. 2017;120:58–65.

[67] Li X, Ma H, Wang Q, Matsumoto S, Maeda T, Ogawa HI. Isolation, identification of sludge-lysing strain and its utilization in thermophilic aerobic digestion for waste activated sludge. Bioresour Technol. 2009;100(9):2475–81. 19147346 10.1016/j.biortech.2008.12.019

[68] Xu Q, Pan W, Zhang R, Lu Q, Xue W, Wu C, et al. Inoculation with Bacillus subtilis and Azospirillum brasilense produces abscisic acid that reduces Irt1-mediated cadmium uptake of roots. J Agric Food Chem. 2018;66(20):5229–36. 29738246 10.1021/acs.jafc.8b00598

[69] He S, Fan X, Luo S, Katukuri NR, Guo R. Enhanced the energy outcomes from microalgal biomass by the novel biopretreatment. Energy Convers Manag. 2017;135(1):291–6.

[70] Wang S, Li J, Liu C, Nies L, Li J. Enhanced methane production through bioaugmentation of butyrate-oxidizing hydrogen-producing acetogens in anaerobic wastewater treatment. Environ Prog Sustain Energy. 2018;37(1):367–74.

[71] Huang C, Wang W, Sun X, Shen J, Wang L. A novel acetogenic bacteria isolated from waste activated sludge and its potential application for enhancing anaerobic digestion performance. J Environ Manage. 2020;255:109842. 10.1016/j.jenvman.2019.109842. 31759203 10.1016/j.jenvman.2019.109842

[72] Shao X, Zhang T, Chen Y, Ai C. Bioaugmentation for improving acidification recovery of an anaerobic sequencing batch reactor after organic shock load. Desalin Water Treat. 2020;185:62–70.

[73] Tale VP, Maki JS, Struble CA, Zitomer DH. Methanogen community structure-activity relationship and bioaugmentation of overloaded anaerobic digesters. Water Res. 2011;45(16):5249–56. 10.1016/j.watres.2011.07.035. 21855955 10.1016/j.watres.2011.07.035

[74] Akila G, Chandra TS. Stimulation of biomethanation by *Clostridium* sp. PXYL1 in coculture with a *Methanosarcina* strain PMET1 at psychrophilic temperatures. J Appl Microbiol. 2010;108(1):204–13. 19566719 10.1111/j.1365-2672.2009.04412.x

[75] Cavaleiro AJ, Sousa DZ, Alves MM. Methane production from oleate: assessing the bioaugmentation potential of *Syntrophomonas zehnderi*. Water Res. 2010;44(17):4940–7. 20696454 10.1016/j.watres.2010.07.039

[76] Wang T, Kuang B, Ni Z, Guo B, Li Y, Zhu G. Stimulating anaerobic degradation of butyrate via *Syntrophomonas wolfei* and *Geobacter sulfurreducens*: characteristics and mechanism. Microb Ecol. 2023;85(2):535–43. 35254501 10.1007/s00248-022-01981-2

[77] Yang Z, Sun H, Zhao Q, Kurbonova M, Zhang R, Liu G, et al. Long-term evaluation of bioaugmentation to alleviate ammonia inhibition during anaerobic digestion: process monitoring, microbial community response, and methanogenic pathway modeling. Chem Eng J. 2020;399:125765.

[78] Zinder SH, Koch M. Non-aceticlastic methanogenesis from acetate: acetate oxidation by a thermophilic syntrophic coculture. Arch Microbiol. 1984;138:263–72.

[79] Zhang Y, Xu Y, Li X, Bai Y, Su Y, Wu D, et al. Exogenous inoculants enhance anaerobic digestion of food and kitchen waste: metabolic and microbial mechanisms. J Environ Chem Eng. 2023;11(6):111251.

[80] Xiao L, Sun R, Zhang P, Zheng S, Tan Y, Li J, et al. Simultaneous intensification of direct acetate cleavage and CO_2_ reduction to generate methane by bioaugmentation and increased electron transfer. Chem Eng J. 2019;378:122229. 10.1016/j.cej.2019.122229.

[81] Yang Z, Wang W, Liu C, Zhang R, Liu G. Mitigation of ammonia inhibition through bioaugmentation with different microorganisms during anaerobic digestion: selection of strains and reactor performance evaluation. Water Res. 2019;155:214–24. 30849735 10.1016/j.watres.2019.02.048

[82] Chen S, He J, Wang H, Dong B, Li N, Dai X. Microbial responses and metabolic pathways reveal the recovery mechanism of an anaerobic digestion system subjected to progressive inhibition by ammonia. Chem Eng J. 2018;350:312–23.

[83] Jain KA, Suryawanshi PC, Chaudhari AB. Hydrogenotrophic methanogen strain of *Methanospirillum* from anaerobic digester fed with agro-industrial waste. Biologia. 2021;76:255–66.

[84] Fotidis IA, Karakashev D, Angelidaki I. Bioaugmentation with an acetate-oxidising consortium as a tool to tackle ammonia inhibition of anaerobic digestion. Bioresour Technol. 2013;146:57–62. 10.1016/j.biortech.2013.07.041. 23916979 10.1016/j.biortech.2013.07.041

[85] Fotidis IA, Karakashev D, Angelidaki I. The dominant acetate degradation pathway/methanogenic composition in full-scale anaerobic digesters operating under different ammonia levels. Int J Environ Sci Technol. 2014;11:2087–94.

[86] Fotidis IA, Treu L, Angelidaki I. Enriched ammonia-tolerant methanogenic cultures as bioaugmentation inocula in continuous biomethanation processes. J Clean Prod. 2017;166:1305–13. 10.1016/j.jclepro.2017.08.151.

[87] Yan M, Treu L, Campanaro S, Tian H, Zhu X, Khoshnevisan B, et al. Effect of ammonia on anaerobic digestion of municipal solid waste: inhibitory performance, bioaugmentation and microbiome functional reconstruction. Chem Eng J. 2020;401:126159. 10.1016/j.cej.2020.126159.

[88] Yan Y, Yan M, Ravenni G, Angelidaki I, Fu D, Fotidis IA. Biochar enhanced bioaugmentation provides long-term tolerance under increasing ammonia toxicity in continuous biogas reactors. Renew Energy. 2022;195:590–7.

[89] Yan M, Zhu X, Treu L, Ravenni G, Campanaro S, Goonesekera EM, et al. Comprehensive evaluation of different strategies to recover methanogenic performance in ammonia-stressed reactors. Bioresour Technol. 2021;336:125329. 34052546 10.1016/j.biortech.2021.125329

[90] Fotidis IA, Wang H, Fiedel NR, Luo G, Karakashev DB, Angelidaki I. Bioaugmentation as a solution to increase methane production from an ammonia-rich substrate. Environ Sci Technol. 2014;48(13):7669–76. 24873631 10.1021/es5017075

[91] Wang H, Fotidis IA, Angelidaki I. Ammonia effect on hydrogenotrophic methanogens and syntrophic acetate-oxidizing bacteria. FEMS Microbiol Ecol. 2015;91(11):fiv130. 10.1093/femsec/fiv130. 26490748 10.1093/femsec/fiv130

[92] Gállego-Bravo AK, García-Mena J, Piña-Escobedo A, López-Jiménez G, Gutiérrez-Castillo ME, Tovar-Gálvez LR. Monitoring of a microbial community during bioaugmentation with hydrogenotrophic methanogens to improve methane yield of an anaerobic digestion process. Biotechnol Lett. 2023;45(10):1339–53. 37535136 10.1007/s10529-023-03414-7 PMC10460350

[93] Hua B, Cai Y, Cui Z, Wang X. Bioaugmentation with methanogens cultured in a micro-aerobic microbial community for overloaded anaerobic digestion recovery. Anaerobe. 2022;76:102603. 10.1016/j.anaerobe.2022.102603. 35709936 10.1016/j.anaerobe.2022.102603

[94] Savant DV, Ranade DR. Application of *Methanobrevibacter acididurans* in anaerobic digestion. Water Sci Technol. 2004;50(6):109–14. 15536997

[95] Town JR, Dumonceaux TJ. Laboratory-scale bioaugmentation relieves acetate accumulation and stimulates methane production in stalled anaerobic digesters. Appl Microbiol Biotechnol. 2016;100:1009–17. 26481626 10.1007/s00253-015-7058-3 PMC4703610

[96] Rinland ME, Gómez MA. Isolation and characterization of onion degrading bacteria from onion waste produced in South Buenos Aires province, Argentina. World J Microbiol Biotechnol. 2015;31:487–97. 25586510 10.1007/s11274-015-1803-8

[97] Jones EJP, Voytek MA, Corum MD, Orem WH. Stimulation of methane generation from nonproductive coal by addition of nutrients or a microbial consortium. Appl Environ Microbiol. 2010;76(21):7013–22. 20817801 10.1128/AEM.00728-10 PMC2976240

[98] Huang L, Liu X, Zhang Z, Ye J, Rensing C, Zhou S, et al. Light-driven carbon dioxide reduction to methane by *Methanosarcina barkeri* in an electric syntrophic coculture. ISME J. 2022;16(2):370–7. 34341507 10.1038/s41396-021-01078-7 PMC8776907

[99] Bagchi S, Behera M. Bioaugmentation using *Pseudomonas aeruginosa* with an approach of intermittent aeration for enhanced power generation in ceramic MFC. Sustain Energy Technol Assess. 2021;45:101138.

[100] Rakic N, Sustersic V, Gordić D, Josijevic M, Jurišević N, Nikolic J. Inoculum to substrate ratio: Calculating methods. 2020.

[101] Tian H, Fotidis IA, Mancini E, Treu L, Mahdy A, Ballesteros M, et al. Acclimation to extremely high ammonia levels in continuous biomethanation process and the associated microbial community dynamics. Bioresour Technol. 2018;247:616–23. 10.1016/j.biortech.2017.09.148. 28985610 10.1016/j.biortech.2017.09.148

[102] Larsen SB, Karakashev D, Angelidaki I, Schmidt JE. Ex-situ bioremediation of polycyclic aromatic hydrocarbons in sewage sludge. J Hazard Mater. 2009;164(2–3):1568–72. 18829166 10.1016/j.jhazmat.2008.08.067

